# Reduced *LHFPL3-AS2* lncRNA expression is linked to altered epithelial polarity and proliferation, and to ileal ulceration in Crohn disease

**DOI:** 10.1038/s41598-023-47997-7

**Published:** 2023-11-22

**Authors:** Katya E. Sosnovski, Tzipi Braun, Amnon Amir, Marina BenShoshan, Haya Abbas-Egbariya, Rakefet Ben-Yishay, Liat Anafi, Camilla Avivi, Iris Barshack, Lee A. Denson, Yael Haberman

**Affiliations:** 1 Sheba Medical Center, Tel-Hashomer, Affiliated with the Tel Aviv University, Tel Aviv, Israel; 2 Faculty of Medicine, Tel Aviv University, Tel Aviv, Israel; 3 Department of Pediatrics, Cincinnati Children’s Hospital Medical Center, University of Cincinnati College of Medicine, Cincinnati, OH USA

**Keywords:** Cell polarity, Gastrointestinal diseases

## Abstract

Disruption of intestinal epithelial functions is linked to Crohn disease (CD) pathogenesis. We identified a widespread reduction in the expression of long non-coding RNAs (lncRNAs) including *LHFPL3-AS2* in the treatment-naïve CD ileum of the RISK pediatric cohort. We validated the reduction of *LHFPL3-AS2* in adult CD and noted a further reduction in patients with more severe CD from the RISK cohort. *LHFPL3-AS2* knockdown in Caco-2 cells robustly affected epithelial monolayer morphogenesis with markedly reduced confluency and spreading, showing atypical rounding, and clumping. mRNA-seq analysis of *LHFPL3-AS2* knockdown cells highlighted the reduction of genes and pathways linked with apical polarity, actin bundles, morphogenesis, and the b-catenin-TCF4 complex. *LHFPL3-AS2* knockdown significantly reduced the ability of cells to form an internal lumen within the 3-dimensional (3D) cyst model, with mislocalization of actin and adherent and tight junction proteins, affecting epithelial polarity. *LHFPL3-AS2* knockdown also resulted in defective mitotic spindle formation and consequent reduction in epithelial proliferation. Altogether, we show that *LHFPL3-AS2* reduction affects epithelial morphogenesis, polarity, mitotic spindle formation, and proliferation, which are key processes in maintaining epithelial homeostasis in CD. Reduced expression of *LHFPL3-AS2* in CD patients and its further reduction with ileal ulceration outcome, emphasizes its significance in this context.

## Introduction

The intestinal epithelium serves as the first line of defense between the luminal microbiota and the host immune cells. The establishment of the apicobasal epithelial barrier polarity is dependent upon fine regulation of the cytoskeletal network, the secretory organelles (ER, Golgi)^1, 2^, and the intracellular trafficking machinery^3^. The three types of cell polarity include the arrangement of molecules through the XY axis, the distribution of molecules around the cell (radial polarity), and the apicobasal polarity on the Z axis^4^. Intracellular trafficking and sorting support the distribution of the polarity domains^5, 6^. Adhesion molecules (cadherins) and tight junction (TJ) proteins establish cellular connections and restrict the diffusion of protein between the apical and basal cell membrane domains^7^. Polarized actin and microtubule cytoskeleton serve as rails for intracellular transport^8–10^ and for orienting organelle positioning, a process that is also required during mitotic spindle formation and cell division^2^. Highly conserved polarity complexes were described; the Crumbs/Pals1/PatJ which mostly localized apically, the Par3/Par6/aPKC/CDC42 that localized at the TJs and is linked with the bipolar attachment of spindle microtubules to kinetochores in metaphase^11^ and is involved in actin cytoskeleton re-organization during cell shape changes^12^, and Scribble (SCRIB))/Dlg/Lgl, which localized at the basolateral surface^4^. Additionally, RAB17 was shown to regulate intracellular membrane trafficking in polarized epithelial cells by recycling endosomes^13^. Failure in sorting and trafficking leads to microvillus inclusion disease, which is linked with severe diarrhea and the inability to absorb nutrients^14^. The disruption of epithelial barrier functions and polarity contributes to abnormal epithelial functions also related to Crohn disease (CD) pathogenesis^3^^,^^15^.

In our previous publication, we identified a widespread reduction in the expression of epithelial long non-coding RNAs (lncRNAs) in treatment naïve CD ileum, including a significant reduction in *LHFPL3-AS2*^16^, a novel relatively uncharacterized lncRNA. LncRNAs are known to regulate fundamental cellular processes, including gene expression, regeneration, and proliferation. This analysis used mRNAseq selection and was limited in its ability to capture non-poly adenylated RNAs^17^. Several lncRNAs are also suggested to regulate epithelial cytoskeleton function^18, 19^ and to modulate critical cell cycle regulators including cyclins, cyclin-dependent kinases (CDKs), and CDK inhibitors^20–24^. Here, we show that the reduction of *LHFPL3-AS2* in Caco-2 cells substantially affects their spontaneous morphology when grown as a monolayer and their ability to form lumens when grown as 3-dimensional (3D) cysts models. This defect is coupled with mislocalization of the actin cytoskeleton and adherent and tight junction proteins, and altered mitotic spindle formation, which results in a reduction in epithelial proliferation.

## Results

### *LHFPL3-AS2* lncRNA is reduced in CD and in patients with more severe mucosal injury

**Figure 1:**
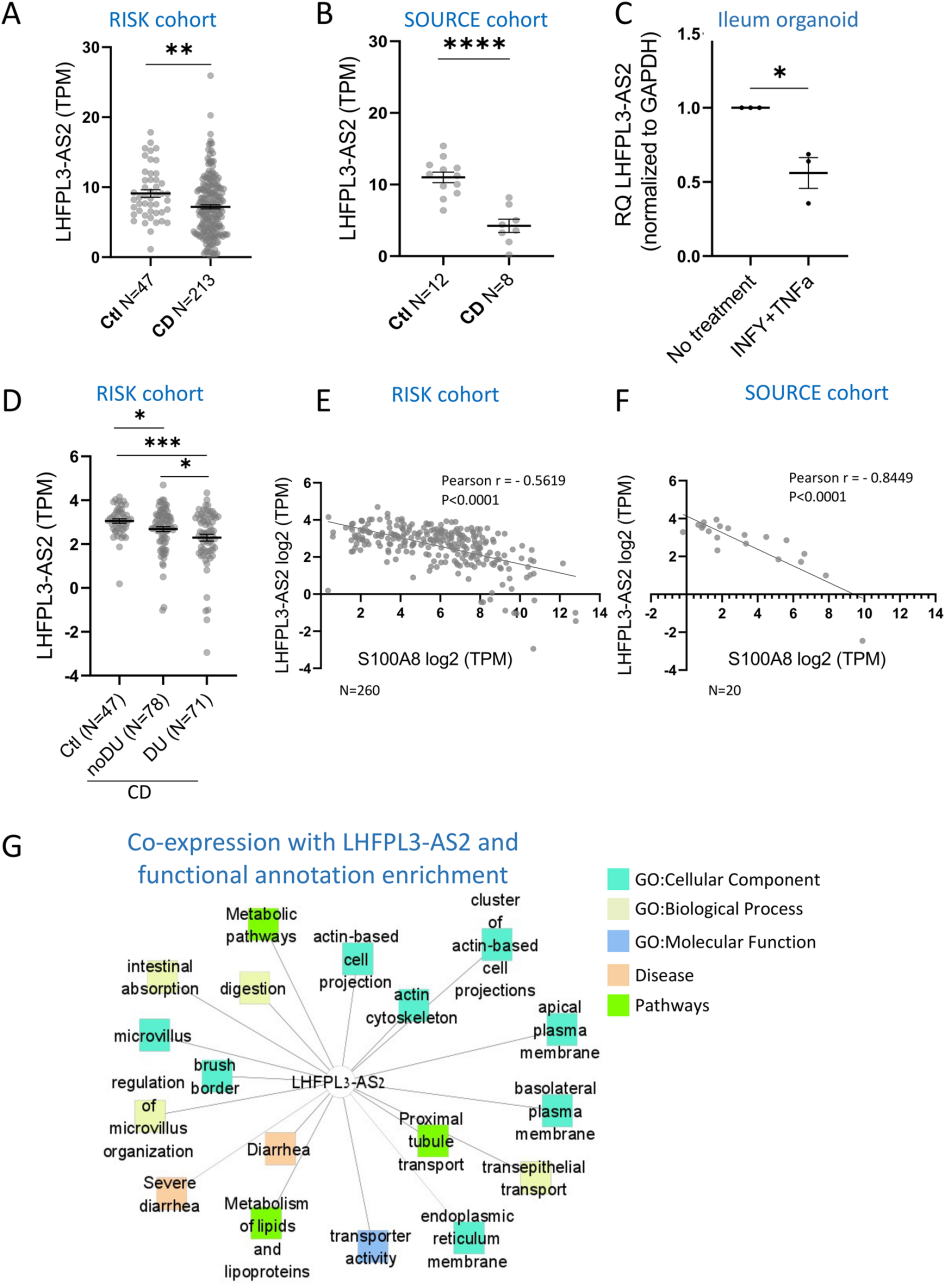
*LHFPL3-AS2* lncRNA is reduced in CD patients and its expression is negatively correlated with CD severity. (**A**,**B**) *LHFPL3-AS2* mRNA is significantly reduced in bulk mucosal biopsies of CD cases vs. controls in two independent cohorts: the pediatric RISK (**A**) 213 CD, and 47 controls) and the adult SOURCE (**B**) 8 CD, and 12 controls) cohorts. (**C**) *LHFPL3-AS2* is expressed in human ileum-derived organoid culture and is reduced upon treatment with 40 ng/ml IFNγ and 20 ng/ml TNFa. (**D**) *LHFPL3-AS2* levels are further reduced in CD patients with deep ulcers (CD-DU, n = 71) in comparison to those without (CD-noDU, n = 78). *LHFPL3-AS2* (TPM) values are shown. (**E**,**F**) *LHFPL3-AS2* log2(TPM) significantly correlates with the calprotectin S100A8 gene as a continuous value in RISK ((**E**), n = 260) and SOURCE (**F**, n = 20). (**G**) Functional annotation enrichment analyses of 470 genes that were co-expressed with *LHFPL3-AS2* in RISK using ToppGene/ToppCluster^32, 33^ and Cytoscape^34^. GO: Biological Process (orange), GO: Cellular component (green), GO: Molecular function (blue). The full list of functional enrichment results and P values are in Supplementary Dataset S1. A two-sided t-test was calculated between groups and Pearson correlation was used for correlations. *P < 0.05, **P < 0.01, ***P < 0.001.

We previously characterized widespread dysregulation of lncRNAs, including *LHFPL3-AS2*, in ileal biopsies of the treatment naïve CD pediatric RISK cohort^16^. We confirmed the reduction of *LHFPL3-AS2* expression in bulk mucosal biopsies of CD patients compared to controls in the RISK cohort (Fig. 1A, p = 0.0083) and validated this reduction also in the adult SOURCE cohort (Fig. 1B, p < 0.0001). Interestingly, significant differences in *LHFPL3-AS2* expression were not detected in celiac disease, another inflammatory disorder affecting the small intestine (duodenum, SEEM cohort, Supplementary Fig. S1). *LHFPL3-AS2* is a relatively uncharacterized lncRNA located on chromosome 7, adjacent and in an antisense orientation to the LHFPL3 protein-coding gene (Supplementary Fig. S1A). While we detected *LHFPL3-AS2* expression in the ileum in both RISK and SOURCE cohorts, *LHFPL3* located in the same locus, showed minimal or no expression in the ileum (Supplementary Fig. S1C) and is therefore unlikely to be regulated in this context by *LHFPL3-AS2*. *LHFPL3* was also not part of the differentially expressed genes between CD and controls in a recent publication^25^*.* Notably, *LHFPL3-AS2* was also expressed in isolated primary epithelia in organoids derived from intestinal biopsies from patients undergoing evaluation via endoscopy, and its expression was significantly reduced upon inflammatory triggering with TNFa plus IFNγ, mimicking the reduction seen in the bulk biopsies in CD patients (Fig. 1C). Secondary analysis of a publicly available mRNAseq dataset ^26^ showed that *LHFPL3-AS2* expression in the ileum of healthy human subjects was significantly higher than in the colon (Supplementary Fig. S1D), and this was also true in isolated epithelia in the ileum vs. sigmoid colon^27^ (Supplementary Fig S1E). The presence of deep ulcers (DUs) in the ileum is known to be associated with endoscopic severity and poorer clinical outcomes^28–30^. Stratifying the RISK CD patients into 2 groups based on the presence or absence of DUs (CD-DU and CD-noDU groups respectively) that was recorded during endoscopy, demonstrated a significant further decrease in *LHFPL3-AS2* levels in those with deep ulcers that represent a more severe endoscopic disease (Fig. 1D). These results are further supported by the significant negative correlation in both the RISK and SOURCE cohorts (RISK: r = − 0.5619, SOURCE: r = − 0.8340, p < 0.0001) between levels of the calprotectin encoding gene (S100A8), which is used as a clinical marker of intestinal inflammation, and *LHFPL3-AS2* expression (Fig. 1E,F). In contrast, similar to previous analyses that showed that clinical severity does not correlate with ileal gene expression^16, 30^ and that clinical severity only poorly correlates with endoscopic severity^31^, no correlation was noted between *LHFPL3-AS2* expression and clinical severity (PUCAI).

To gain further insights into the potential functions of *LHFPL3-AS2*, we analyzed its co-expression within the previously defined core CD signature in RISK^16^ (2160 protein-coding genes). We identified 470 genes with similar patterns of expression as *LHFPL3-AS2* (Pearson correlation, 0.98 < r < 1) across the predefined patient groups (controls, CD-DU, and CD-noDU) with a high likelihood for coregulation and shared biological function. Functional annotation enrichment analyses of these genes using ToppGene^32^ (Fig. 1G and Supplementary Dataset S1) showed enrichment for terms related to the actin cytoskeleton (FDR P < 1.66E − 07), apical (FDR P < 2.97E − 20) and basal membrane (FDR P < 1.80E − 05), endoplasmic reticulum (ER) membrane (FDR P < 2.33E − 03), transepithelial transport (FDR P < 2.78E − 05), microvillus (FDR P < 3.88E − 15) and intestinal absorption (FDR P < 1.24E − 05). Disease-enriched terms included diarrhea (FDR P < 5.47E − 04) and gastrointestinal diseases (FDR P < 3.17E − 02).

### *LHFPL3-AS2* knockdown interrupts Caco-2 morphology

Comparing the CD cohorts and Caco-2 mRNAseq analyses indicated that many of the epithelial lncRNAs that were reduced in CD, including *LHFPL3-AS2*, are expressed in Caco-2 cells^16^. Caco-2 cells are human-derived colon epithelial cancer cells that can be grown as a polarized monolayer expressing a phenotype resembling that of intestinal enterocytes including tight junctions and metabolizing enzymes^35^ or as a 3-dimensional (3D) polarized cysts model^36^. We, therefore, used this cell line as a convenient and robust system to characterize *LHFPL3-AS2* and to test the effect of *LHFPL3-AS2* loss of function (LOF) on epithelial functions. We first confirmed that *LHFPL3-AS2* expression in this system shows a similar pattern as in mucosal biopsies enriched for epithelia using mRNAseq (Supplementary Fig. S2A). Fractionation showed that *LHFPL3-AS2* is distributed between the nucleus and the cytoplasm (Supplementary Fig. S2B), and fluorescence RNA in situ hybridization (FISH) showed *LHFPL3-AS2* probes localizing to perinuclear and nuclear regions. Since *LHFPL3-AS2* cytoplasmic distribution was adjacent to the nucleus, which is the approximate location of the ER, and since in the co-expression analysis of the human cohort we observed an enrichment in ER-related genes, we assessed co-localization of* LHFPL3-AS2* with the ER marker calnexin (CANX) and observed substantial overlap (Supplementary Fig. S2D).

**Figure 2:**
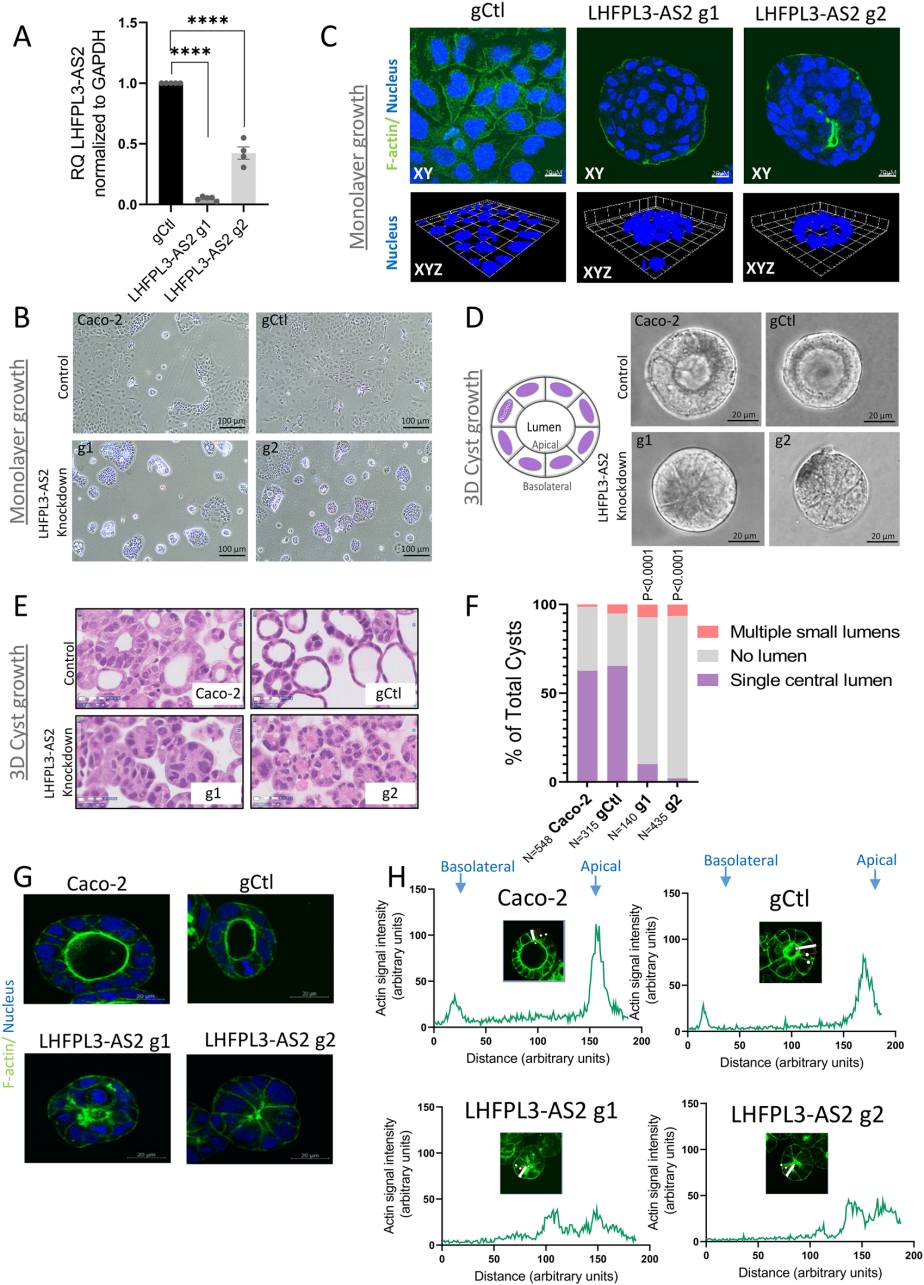
*LHFPL3-AS2* knockdown results in robust loss of Caco-2 luminal cyst formation. (**A**) *LHFPL3-AS2* knockdown was achieved using CRISPRi and 2 gRNAs, and those cells were compared with gCtl. qPCR confirmed a reduction in *LHFPL3-AS2*, after normalization to GAPDH. The two-sided t-test is shown. ****P < 0.0001. (**B**) Light microscopy imaging (× 10) of *LHFPL3-AS2* knockdown or Caco-2 and gCtl cells after seeding them as a monolayer on a culture plate for 3 days, showing spontaneous rounding and clumping in the knockdown cells. Scalebar—100uM, magnification – × 10. (**C**) F-actin Phalloidin staining (green) and nuclear staining (blue) of *LHFPL3-AS2* knockdown or gCtl cells after seeding them as a monolayer on a culture plate. The upper panel shows the xy-axis views and the lower panel shows a 3D reconstruction of z-stack images. *LHFPL3-AS2* knockdown cells show a rounding appearance as demonstrated on the z-axis when grown as a monolayer, with less confluency and more clumping. Magnification x63oil, scalebar-20 µM. (**D**) Schematic representation and bright field imaging of *LHFPL3-AS2* knockdown or Caco-2 and gCtl 3-dimensional (3D) cyst grown in Matrigel for 5 days, with lumen/apical part inside. Scalebar—20uM, magnification – × 40. (**E**) Haematoxylin and eosin (H&E) staining of Caco-2 cysts shows that most *LHFPL3-AS2* knockdown cysts have no lumen. Scalebar—20uM, magnification – × 40. (**F**) Quantification of (**D**) indicating the percentage of cysts with a single central lumen, no lumen, or multiple lumens were quantified. Fisher exact test was performed comparing the number of cysts with a single lumen to those with no lumen or with multiple small lumens. (**G**) Phalloidin staining for F-actin (green) and nuclear Hoechst (blue) staining of Matrigel-grown cysts. Magnification x63oil, scalebar-20uM. (**H**) Line plots denoting the intensity of actin staining along the apical/basal axis of indicated cyst along the white line, from the apical to basolateral side. The image shows a representative experiment out of 6 replications (shown in Supplementary Fig. S3).

We developed a loss of function (LOF) system to mechanistically model our observations of *LHFPL3-AS2* reduction in CD patients. Using CRISPRi and two independent *LHFPL3-AS2*-specific guide RNAs (g1 and g2 gRNAs, Supplementary Table S2), we depleted *LHFPL3-AS2* expression as confirmed by qPCR in comparison to control gRNA (gCtl, Fig. 2A). *LHFPL3-AS2* knockdown cells (with either g1 and g2) exhibited abnormal morphology when grown as monolayer (Fig. 2B,C), showing markedly reduced confluency and spreading on the plate, with atypical rounding and clumping that was detected more substantially on the Z-axis (Fig. 2C). This atypical rounding of *LHFPL3-AS2* knockdown cells when cells were simply seeded as a monolayer, and the lack of cells confluency hampered our ability, for example, to measure trans-epithelial electrical resistance (TEER) or permeability in the knockdown cells. We, therefore, looked for alternative growth conditions in 3D that will enable head-to-head comparison of *LHFPL3-AS2* knockdown and controls. We turned to the Caco-2 cysts 3-dimensional model to systematically test the effects of *LHFPL3-AS2* knockdown. In this system, when Caco-2 cells are cultured in Matrigel, they form lumenized 3D cysts^36^ in which the inside layer is the apical luminal membrane, and the outer membrane represents the basolateral side (Fig. 2D). Bright field imaging (Fig. 2D) and hematoxylin and eosin (H&E, Fig. 2E) staining of the fixed 3D cyst cultures showed that *LHFPL3-AS2* knockdown cells failed to form internal luminal cavities. Systematic quantification of cyst morphology indicated that only 10% of *LHFPL3-AS2* knockdown cysts formed internal lumens, compared to 62–65% of control cysts (Fig. 2F, p < 0.0001). In addition, F-actin staining showed the expected dominant apical localization in control cysts, whereas in *LHFPL3-AS2* knockdown cysts, F-actin was spread throughout the cell membrane (Fig. 2G). We also measured the F-actin intensity signal peaks along the apical-basolateral axis of the 3D cysts as previously described^37^, which further highlighted the abnormal apical: basolateral distribution of actin in *LHFPL3-AS2* knockdown cells (Fig. 2H, Supplementary Fig. S3). Moreover, our microscopic analyses showed internal inclusions of apical markers in *LHFPL3-AS2* knockdown cysts Fig. 2F and Supplementary Figs. S3 and S4). This pattern resembles the result of failed protein trafficking, as seen in microvillus inclusion disease, which leads to severe diarrhea and inability to absorb nutrients^38^.

### *LHFPL3-AS2* knockdown results in a robust reduction in the expression of genes involved in epithelial morphology and structure

**Figure 3:**
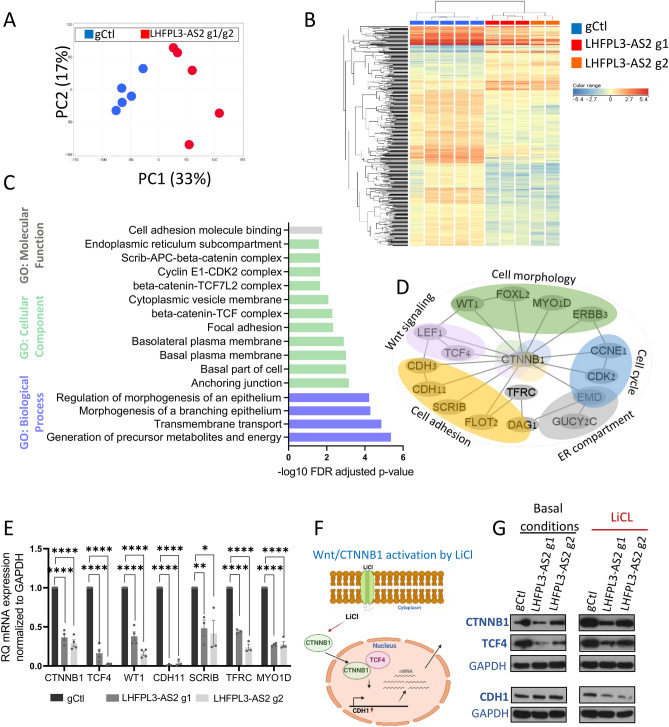
*LHFPL3-AS2* knockdown reduced expression of genes, known to regulate epithelial morphology, including CTNNB1. (**A**) PCA using 11,489 protein-coding genes that passed expression filtering and colored by *LHFPL3-AS2* knockdown (g1 and g2 in red) and gCtl (blue). (**B**) Heatmap of 584 genes that were differentially expressed between *LHFPL3-AS2* knockdown cells (g1 and g2) compared to controls (gCtl, fold change >  = 1.5 and FDR corrected p <  = 0.05). (**C**) ToppGene^32^ functional annotation enrichment of 462/584 downregulated gene protein-coding genes with the associated − log10(FDR P value). (**D**) STRING^43^ predicted interaction network with visualization using Cytoscape^34^ of the downregulated genes. (**E**) qPCR validation of genes that were reduced in the mRNAseq dataset, normalized to GAPDH. The two-sided t-test is shown. *P ≤ 0.05, **P ≤ 0.01, ***P ≤ 0.001, ****P ≤ 0.0001. (**F**) Schematic representation of Wnt signaling activation by LiCl. (**G**) Western blots (representative experiment out of 3 replications) of *LHFPL3-AS2* knockdown cells (g1 and g2) and controls (gCtl) with and without LiCl to activate the Wnt pathway showing reduction of CTNNB1 and TCF4 at baseline and after LiCl in *LHFPL3-AS2* knockdown cells, and of CDH1 with LiCl.

We performed mRNASeq analysis of *LHFPL3-AS2* knockdown cells and controls that were seeded as monolayers. Principal component analysis (PCA) using all genes that passed expression filtering (n = 11,489) showed a clear separation between the knockdown cells (g1 and g2) and control (Fig. 3A). We identified 584 genes that were differentially expressed between the *LHFPL3-AS2* knockdown cells compared to control cells (fold change >  = 1.5 and FDR corrected p <  = 0.05; Fig. 3B), with the majority of the genes (462/584; 79%) being downregulated upon *LHFPL3-AS2* knockdown. Among the reduced genes we found several key regulators of polarity processes, including, for example, RAB17 [fold change (FC) = − 3.2], a GTPase which functions as a regulator of intracellular membrane trafficking primarily in epithelia^13^, *CDC42EP3* (FC = − 3.6)*,* which is involved in actin cytoskeleton re-organization downstream of *CDC42*^39^ to induce actin filament assembly leading to cell shape changes, SCRIB (FC = − 6.5), which involved in the establishment of apicobasal cell polarity^40, 41^, and BMP family genes, which are pivotal for maturation and differentiation of intestinal epithelial cells^42^. Functional enrichment of the 462 downregulated genes (Fig. 3C and Supplementary Dataset S2) showed enrichment for terms including basal part of the cell (FDR P = 0.00002534) and basal plasma membrane (FDR P = 0.00002534), endoplasmic reticulum compartment (FDR P = 0.02614), transmembrane transport (FDR P = 0.00001377), anchoring junction (FDR P = 0.0006941), regulation of morphogenesis of an epithelium (FDR P 0.00006018, 0.00005167), cell adhesion molecule binding (FDR P = 0.01726), b-catenin-*TCFL2* complex (FDR P = 0.02249) and cyclin E1-CDK2 complex (FDR P = 0.02249).

Using String, a functional protein association networks tool, we connected some of the downregulated genes to β-catenin (*CTNNB1*), which was identified as a central node in this sub-network (Fig. 3D). We validated the reduced expression of *CTNNB1, TCF4, WT1, CDH11, SCRIB, TFRC,* and *MYO1D* in *LHFPL3-AS2* knockdown cells by qPCR (Fig. 3E), and the reduction in CTNNB1 and TCF4 at the protein level using western blotting (Fig. 3G). CTNNB1 and TCF4 are part of the canonical Wnt signaling pathway, which plays a role in the development and renewal of the intestinal epithelium; WT1 and MYO1D are involved in cell morphology while CDH11 and SCRIB are part of the adhesion process. Next, we stimulated Caco-2 cells with Lithium chloride (LiCl), which is known to activate Wnt/β-catenin signaling by inhibiting the GSK3 activity^37, 44^, thereby increasing the free β-catenin fraction that translocates to the nucleus to activate the TCF4 transcription factor and its downstream genes (Fig. 3F scheme). A similar reduction was noted in *TCF4* and *CTNNB1* levels in *LHFPL3-AS2* knockdown cells at baseline and after LiCl treatment, but we also documented a reduction in E-cadherin (CDH1) more persistently upon LiCl treatment. CDH1 is a known adherens junction protein and a downstream target of CTNNB1 (Fig. 3G)^45, 46^. We next examined the distribution of tight and adherens junction proteins by immunofluorescence in Caco-2 3D cysts. Staining for these markers showed that in knockdown cells, most were abnormally distributed (Supplementary Fig. S4). Specifically, the apical markers F-actin and ZO-1 were expressed in a non-homogenous pattern with intracellular vesical-like structures in cysts that did not form lumens, and the junction marker CDH1 similarly localized intracellularly in clusters in *LHFPL3-AS2* downregulated cells (Supplementary Fig. S4A). We also captured reduced *ZO-1* and E-cadherin *CDH1* mRNA levels in *LHFPL3-AS2* downregulated cells (Supplementary Fig. S4B-C). Staining for the intercellular junctional markers CTNNB1 and JAM-A also showed disorganized phenotypes in *LHFPL3-AS2* down-regulated cysts (Supplementary Fig. S4D).

### *LHFPL3-AS2* downregulation induces abnormal mitotic divisions with multipolar spindles, and reduced proliferation

**Figure 4:**
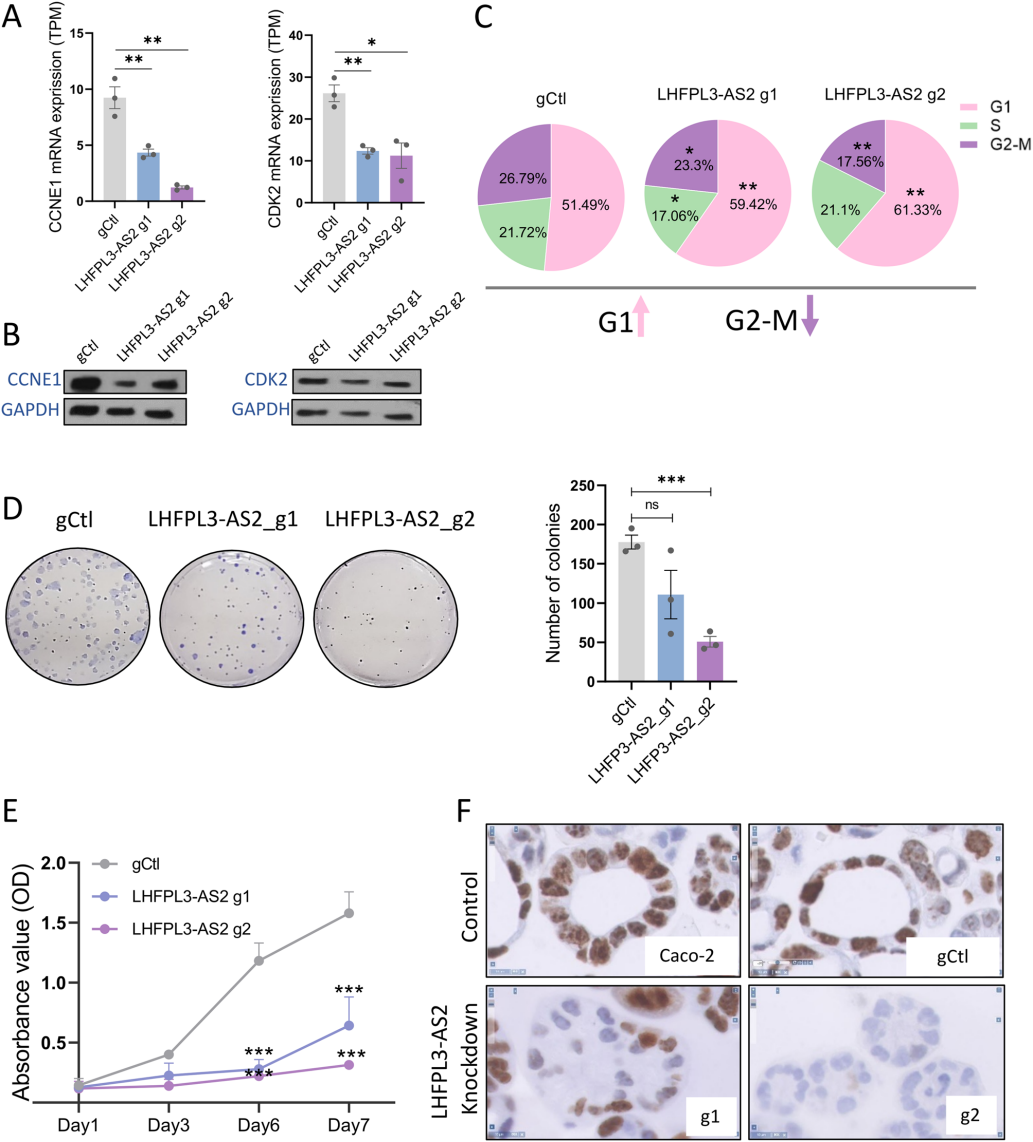
*LHFPL3-AS2* downregulation inhibited cell proliferation resulting in G1 cell cycle arrest. (**A**) mRNA levels (TPM) of CCNE1 and CDK2 in *LHFPL3-AS2* knockdown cells (g1 and g2) and controls (gCtl). (**B**) Western Blot analysis of CCNE1 and CDK2 using GAPDH as a loading control (representative experiment out of 3 replications). Quantifying the protein expression, using ImageJ, and normalizing it to GAPDH indicated a reduction in CCNE1 of 59% with g1 and 24% with g2 and a more modest reduction in CDK2 of 16% with g1 and 18% with g2. (**C**) Propidium Iodide staining (PI) and FACS analyses of *LHFPL3-AS2* knockdown cells (g1 and g2) and controls (gCtl). Percentages of cells in G1, S, and G2/M cell-cycle phases are in a pie chart. One sided t-test, n = 4, *p < 0.05, **p < 0.01. (**D**) Colony formation assay with representative images (left panel) and quantification (right panel) of the colonies (n = 3). (**E**) XTT assay absorbance values (OD) (n = 3) show that suppression of *LHFPL3-AS2* inhibited Caco-2 cell proliferation. (**F**) Immunohistochemistry (IHC) staining of Ki-67 in paraffin-embedded sections of Caco-2 3D cysts. Scalebar – 10uM, magnification – × 40.

RNAseq analyses of *LHFPL3-AS2* knockdown cells further indicated significant downregulation of cyclin E1 *(CCNE1)* and* CDK2*, a complex that is required for cell cycle G1/S transition (Supplementary Dataset S2 and Fig. 4A), and we validated the reduction in CCNE1 and CDK2 at the protein level (Fig. 4B). This observation, together with the known role of CTNNB1 and Wnt in regulating proliferation, prompted us to explore whether *LHFPL3-AS2* affects cell division. Indeed, *LHFPL3-AS2* knockdown resulted in an increase in the cell fraction arrested at the G1 phase, with subsequent reduction in G2/M (Fig. 4C and Supplementary Fig. S5 showing representative FACS results). The G1 arrest in *LHFPL3-AS2* knockdown cells led to attenuated cell proliferation as measured by decreased colony formation (Fig. 4D), and XTT assay (Fig. 4E) in monolayer cells, as well as Ki67 staining, a nuclear protein that is associated with cellular proliferation, in cysts (Fig. 4F).

**Figure 5:**
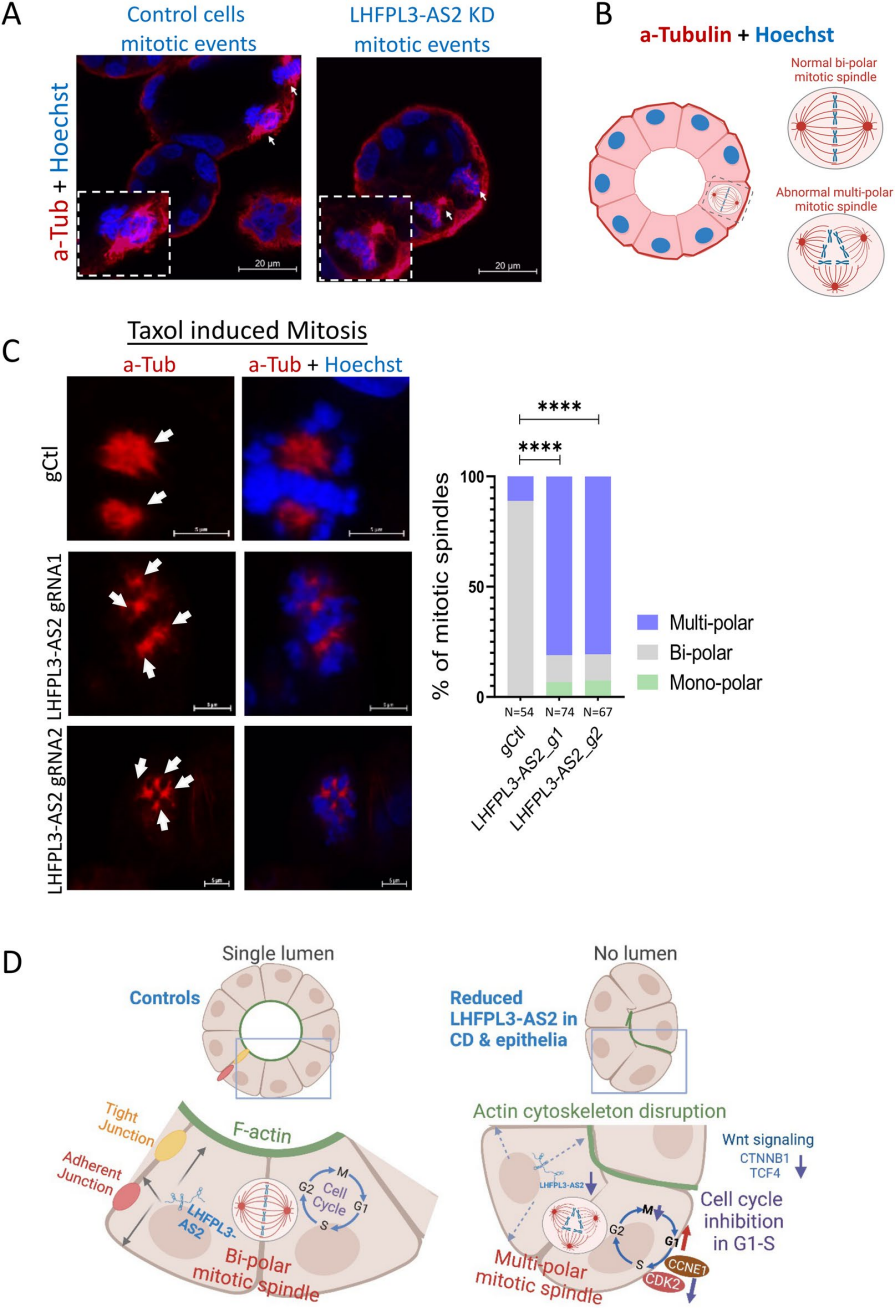
*LHFPL3-AS2* knockdown results in abnormal multipolar mitotic spindle formation. (**A**) Fluorescence staining using anti-tubulin (red) and nuclear Hoechst (blue) of *LHFPL3-AS2* CRISPRi knockdown and gCtl Caco-2 cysts. White arrows indicate mitotic spindles. Scalebar—20uM, magnification—x60oil. (**B**) Schematic representation of a normal bipolar mitotic spindle. (**C**) Caco-2 grown as 3D cysts were treated with 20 nM Taxol for 24 h to arrest cells during mitosis. Left panel—representative images of the mitotic spindles in *LHFPL3-AS2* knockdowns and controls (gCtl). White arrows indicate centrosomes. Right panel—Percentage of cells with bipolar spindles, multipolar spindles, or monopolar spindles captured during mitosis. Fisher exact test between the fraction of cells with bi-polar spindles vs. multi/mono-polar spindles. ****P ≤ 0.0001. (**D**) Schematic cartoon summarizing *LHFPL3-AS2's* potential role in regulating epithelial polarity, proliferation, and mitotic spindle formation. Reduction of *LHFPL3-AS2* affects apicobasal positioning of cytoskeleton protein (actin) and tight/adherens junction proteins. Loss of cellular polarity and abnormal cellular positioning likely underlies the abnormal mitotic spindle and centrosome arrangement, resulting in G1 cell cycle arrest, reduced cellular proliferation, and the robust subsequent inhibition of transcription and translation of many key epithelial genes including *CTNNB1 and TCF4*.

Cell cycle arrest has been linked with several centrosome defects, such as centriole loss, separation, and fragmentation^47^. This together with the effect of *LHFPL3-AS2* reduction on cyst lumen formation, epithelial polarity, and cell proliferation suggested that *LHFPL3-AS2* loss may affect a mitotic and cell division rearrangement. By staining for α-tubulin and DNA (Hoechst), we were able to capture several mitotic events in Caco-2 cysts. Whereas in the control cysts we observed the normal bipolar mitotic spindle, in *LHFPL3-AS2* knockdown cysts we noted a disorganized multipolar pattern (Fig. 5A and B). Aiming to capture more mitotic events, we used Taxol (Paclitaxel, 20 nM for 24 h), which stabilizes microtubules and reduces their dynamics in the G2/M phase, resulting in mitotic arrest^48^. Upon Taxol treatment, *LHFPL3-AS2* knockdown resulted in a significant increase in multipolar spindle formation and centrosome amplification (Fig. 5C, white arrows, Supplementary Fig. S6). Indeed, 80–81% of cells in *LHFPL3-AS2* knockdown cysts showed multipolar spindles compared to just 11% of control cells (Fig. 5C, Supplementary Videos 1–3).

## Discussion

The apical and basal plasma membrane domains are differentially equipped with proteins to control the absorption and secretion of nutrients between the gut lumen, cell interior, and the intestine. Defects in apicobasal polarity disrupt the epithelial barrier defense against pathogens and affect cellular metabolism and uptake of nutrients^49^, and disruption in these epithelial functions contributes to CD pathogenesis^3^. Herein, we highlight a significant reduction of *LHFPL3-AS2*^16^, a novel, relatively uncharacterized lncRNA, in two independent CD datasets (RISK, SOURCE). In addition, we show that *LHFPL3-AS2* is further downregulated in patients with more severe disease, as determined by the presence of deep ulcers, and with higher mucosal calprotectin, a marker of intestinal inflammation assessment used in clinic, mRNA levels. To identify potential *LHFPL3-AS2* functions, we performed co-expression analysis ("guilt-by-association") in the human cohort^50^, and showed enrichment for genes linked with the actin cytoskeleton, apical and basal membranes, and the ER sub-compartment. We demonstrated that *LHFPL3-AS2* is predominantly expressed in the small rather than large intestine, is distributed between the nucleus and cytoplasm, and colocalizes with the ER marker Calnexin in Caco-2 cells.

Depletion of *LHFPL3-AS2* in Caco-2 cells resulted in a robust spontaneous alteration of cellular morphology when cells were grown as a monolayer. *LHFPL3-AS2*-depleted cells rounded up and clumped and for that reason, we could not reach cellular confluency and were unable to measure for example TEER or permeability. Since *LHFPL3-AS2* knockdown demonstrated a more 3D structure, it encouraged us to turn to the more challenging 3D cyst-polarized Caco-2 model for imaging and quantification of the cellular abnormalities of both the knockdowns and the controls in the same conditions. Using the 3D cyst-polarized model we were able to show substantial and significant defective lumen formation and altered actin distribution at the apicobasal axis. Cell rounding and insufficient spreading may interpret as diminished cell-extracellular matrix interactions, but the defects seen in the lumen formation in 3D, more likely suggest an abnormal polarity. Respectively, we observed that *LHFPL3-AS2*-depleted cysts retained internal inclusions of apical markers (i.e., actin). This intracellular vesicular pattern suggests a failure of protein trafficking, rather than pure differentiation defects, which is also observed in polarity-associated disorders like microvillus inclusion disease (MVID) that leads to severe diarrhea and inability to absorb nutrients in the intestine. Diarrhea and poor absorption are also key components of CD pathogenesis. Moreover, proper epithelial renewal and proliferation is part of the mucosal healing process, which is the main treatment goal in CD^51^, and our results suggest that this process is also attenuated by *LHFPL3-AS2* knockdown, which can lead to abnormal healing.

Differential RNAseq expression analysis further supported this concept. We identified a reduction of 462 genes in *LHFPL3-AS2*-depleted cells including several that are known to have key roles in cellular polarity, such as RAB17, an epithelial cell-specific GTPase^13^, and *CDC42EP3,* which acts downstream of *CDC42*^39^ to induce actin filament assembly leading to cell shape changes, BMP family proteins which affect cell differentiation^42^, and SCRIB which regulates the establishment of apicobasal cell polarity^40, 41^ and the progression from G1-S cell cycle phase^41, 52^. Functional enrichment of the reduced genes highlighted pathways linked with the apical cell membrane, actin filament bundle, microvillus, regulation of morphogenesis of epithelium, adherens junctions, β-catenin-*TCF4* complex, and connection to the ER compartment. Membrane proteins are synthesized in the ER and transferred to the trans-Golgi network, where they are sorted into distinct endosomal carrier vesicles and transported to the apical or basolateral plasma membrane^53^. Failure of vesicle trafficking causes apical proteins to accumulate sub-apically and can result in microvillus inclusion disease^14^. Previous studies have indicated that assembly of the E-cadherin/β-catenin complex occurs in the very early stage of the intracellular trafficking^54–56^, and that this process is the obligatory step in efficient export from the ER and for subsequent delivery of E-cadherin to the basal-lateral membrane of polarized MDCK cells^56^. We identified *CTNNB1* as a central node in the network of reduced genes resulting from *LHFPL3-AS2* knockdown. The overall effect observed from the reduction of *LHFPL3-AS2* knockdown is summarized in Fig. 5D; the reduction of *LHFPL3-AS2* affects epithelial polarity and apicobasal positioning of cytoskeleton protein (actin) and tight/adherens junction proteins. Loss of cellular polarity and abnormal cellular positioning likely underlies the abnormal mitotic spindle and centrosome arrangement, resulting in G1 cell cycle arrest, reduced proliferation, and subsequent inhibition of transcription and translation of many key epithelial genes including *CTNNB1* and *TCF4*. *LHFPL3-AS2* may participate in regulating intracellular trafficking and epithelial polarity through its cytoplasmic ER localization, but further studies are needed to support this idea.

Cell division provides a mechanism for maintaining the structural integrity of the epithelial barrier in rapidly proliferating tissues, such as the intestine^57^. Notably, centrosome duplication, the initial event in cell division, takes place during G1–S transition and defects in centrosome positioning can disrupt epithelial homeostasis^58^. *LHFPL3-AS2* silencing resulted in reduction in CCNE1 and CDK2, which regulate the transition from G1 to S phase^59^. We also noted that *LHFPL3-AS2* knockdown cysts displayed multipolar spindle formation and abnormal centrosome integrity. Similar to the effects seen here, ZO-1-deficient colonic epithelia have aberrantly oriented mitotic spindles, resulting in abortive proliferation^60^. Loss of centrosome integrity was recently shown to trigger checkpoint regulators that inhibit G1–S progression^47^, and cells arrested in the G1 phase can assemble more than four centrioles^61^. Other lncRNAs like *gadd7*, which destabilizes *CDK6* mRNA^21^, were previously shown to control cell cycle progression via modulation of the expression of critical cell cycles regulators such as cyclins, CDKs, and CDK inhibitors^20–24^, but here we anticipate inhibition of proliferation and the G1 arrest may be secondary to improper epithelial polarity linked with abnormal spindle positioning. *LHFPL3-AS2* is a relatively uncharacterized lncRNA, with one recent publication indicating that it suppresses metastasis of non-small cell lung cancer (NSCLC) by interacting with *SFPQ* to regulate *TXNIP* expression^62^. Our RNA-seq analyses however identified *TXNIP* to be up-regulated upon *LHFPL3-AS2* reduction. Importantly, over-expression of *TXNIP* (as seen here as a result of *LHFPL3-AS2* reduction in Caco-2 cells) is known to induce G0/G1 cell cycle arrest^63^ which is consistent with our observation. A possible explanation for discrepancies between these studies may be that lncRNAs have different expression profiles and mechanisms of action in different cell types. For instance, *HNF1A-AS1* lncRNA has been defined as a tumor suppressor gene in laryngeal squamous cell carcinoma^64^, but also has an oncogenic role in gastric cancer^65^. It is also possible that regulatory RNAs such as miRNA and circRNA mediate the effect seen upon *LHFPL3-AS2* reduction, and this concept will need to be further explored.

A notable strength of our study was the use of treatment-naïve adult and pediatric patient cohorts, free from confounding variables of previous therapy. We used diverse, complementary wet-lab and mRNAseq informatics approaches to cross-validate our results, which altogether support a model whereby *LHFPL3-AS2* regulates intestinal epithelial functions. However, our use of treatment-naïve samples is also a limitation, as the associated inflammation may confound results. Additionally, for studying the effect of *LHFPL3-AS2* knockdown, we used a tissue culture model rather than an animal model or organoid system. However, human-derived cell lines are widely used in recently published work to understand the role of long non-coding RNAs (lncRNAs) in human-related systems^66^^,^^67^ and functions. Although Caco-2 cells have hyper-tetraploid karyotypes, we showed that in most of the cases (89%) their mitotic spindles had a normal bipolar phenotype, unlike the *LHFPL3-AS2* 3D cysts. Furthermore, we confirmed that Caco-2 cells show a similar pattern of *LHFPL3-AS2* expression as that seen in mucosal biopsies enriched for epithelia, indicating that it is an appropriate model system. Finally, while we saw a robust effect on spontaneous cellular morphology on cells grown as monolayer and in 3D polarized cyst culture when *LHFPL3-AS2* was inhibited by 2 independent gRNAs, we did not fully explore the mechanism by which *LHFPL3-AS2* regulates those functions. However, RNAseq data of the knockdown cells did show a significant and substantial reduction in polarity-associated genes (SCRIB was reduced with a fold change (FC) =  − 6.5, CDC42EP3 FC =  − 3.6, and RAB17 with FC =  − 3.2). *LHFPL3-AS2* rescue experiments are valuable to confirm *LHFPL3-AS2* role but will be part of future work. It will also be interesting to examine if CTNNB1 over-expression, which is a predicated candidate of the enrichment analyses of the *LHFPL3-AS2* knockdown*,* is able to rescue the LHFPL3-AS2 phenotypes.

In conclusion, we show that *LHFPL3-AS2* controls epithelial morphogenesis by regulating epithelial polarity, mitotic spindle formation, and proliferation. Epithelial polarity and proliferation are key in maintaining epithelial homeostasis and functions that are relevant to CD. Reduced expression of *LHFPL3-AS2* in CD and its further reduction in more severe CD forms emphasizes the clinical significance in this context. Future studies are needed to determine how *LHFPL3-AS2* regulates epithelial polarity potentially via its localization to the ER.

## Materials and methods (see supplementary methods)

### Cohorts, study approval, and RNAseq analyses

We performed secondary analyses of the published pediatric RISK (GSE101794) and the adult SOURCE (GSE199906) treatment naïve CD cohorts to test the baseline expression of *LHFPL3-AS2* in CD in comparison to controls in the ileum. Secondary analyses of other published cohorts were used for expression validation of *LHFPL3-AS2* in the ileum and colon tissue in healthy controls in bulk biopsies^26^, and in isolated epithelia^26^. Sheba Medical Center Institutional Review Board approved the SOURCE CD cohort (Supplementary Table S1) protocol and informed consent was obtained. *LHFPL3-AS2* expression was assessed after uniformly reanalyzing the transcriptomics raw FATSQ files. RNA-seq on *LHFPL3-AS2* knockdown Caco-2 cells was performed using Lexogen QuantSeq 3' mRNA-Seq libraries sequencing (data was deposited in GEO: GSE216810). Principal Coordinates Analysis (PCA) was performed to summarize differences between knockdown and control conditions. In downstream analysis, 11,489 protein-coding mRNA genes with Reads per Milion (RPM) above 3 in 20% of the samples were included. Differentially expressed genes were determined in GeneSpring^®^ software with fold change differences (FC) ≥ 1.5 and using the Benjamini–Hochberg false discovery rate correction (FDR ≤ 0.05). ToppGene^32^, ToppCluster software were used to perform functional annotation enrichments, and Cytoscape.v3.0.2^68^ was used for visualization.

### *LHFPL3-AS2* knockdown in cell culture

*LHFPL3-AS2* stable knockdown was achieved by infecting Caco-2 cells with CRISPR/dCAS9-KRAB inactivator plasmid that was packaged into virions generated in HEK293T cells using a lentiviral vector system. Monoclonal clones were selected with Puromycin. Then, the pKLV-gRNAs clones which contained the U6 promoter and hygromycin gene (hygro) were packaged into virions and generated in HEK293T cells using a lentiviral vector system. Afterward, a stable clone of Caco-2 expressing CRISPRi plasmid was infected by a lentivirus carrying the specific gRNA or by an empty plasmid that is used as a control (gCtl). gRNA sequences and qPCR primers are in Supplementary Tables S2 and S3 respectively.

### Confocal immunofluorescence

Cells were grown as monolayers on coverslips in 12 well plates, or Matrigel in 24 well plates for 3D cysts, fixed with 4% paraformaldehyde, permeabilized with 0.1% Triton X-100 and blocked in 5% BSA. Then, the cells were incubated with primary antibodies diluted in 5% BSA overnight at 4C, the coverslips were fluorescently labeled and incubated with a secondary antibody (anti-rabbit or anti-mouse Cy3 or Cy5; Abcam). For visualization of F-actin, Phalloidin (in 5% BSA; Abcam) staining was performed for 20 min, followed by Hoescht staining (Sigma). The coverslips were examined using Zeiss confocal microscope (Carl Zeiss AB, Stockholm, Sweden). Images were processed using ZEN 3.1 (blue edition) browser software. For the 3D system, images were produced using a Z-stack confocal-based scan of the cysts. Movies were produced from Z-stack images using the ZEN 3.1 (blue edition) browser. For mitotic spindle visualization, Caco-2 3D cysts were treated with 20 nM Taxol for 24 h and stained with a-Tubulin. Mono/bi/multi-polar spindles were quantified manually, across the Z-stack of the cyst. Apical-basal actin intensity was determined, using the profile feature, within the ZEN 3.1 software, which shows intensity to distance curve of the region of interest (luminal apical to the basolateral side of the cyst). For RNA fluorescent in situ hybridization (FISH), Caco-2 cells were seeded on 18 mm glass coverslips in a 12-well plate. Stellaris RNA FISH was used to visualize *LHFPL3-AS2* and CANX antibody was used for co-localization with ER, the cells were fixed and stained according to Stellaris protocol (Biosearch Technologies). Confocal imaging was performed with a Zeiss confocal microscope using a 63 × oil immersion objective.

### Proliferation assays

For colony formation assay, Caco-2 cells were seeded (1,000 cells/6 well plate) and cultured for 13 days to form colonies, fixed with 100% ethanol, and stained with 10% Giemsa, photographed, and counted. For cell viability assay, Caco-2 cells were seeded into a 96-well plate, and proliferation was assessed using the XTT Cell Proliferation Assay Kit (Biological Industries) following the manufacturer's protocols. For proliferation assessment in 3D cysts, paraffin-embedded sections of the cysts were stained with Ki67 antibody.

### Fluorescence-activated cell sorting (FACS) cell cycle analyses

Cells were fixed in 70% ice-cold ethanol at 4 °C and stained with 5 mg/ml propidium iodide (Sigma) Fluorescence intensity was analyzed using Navios flow cytometer (Beckman Coulter). The analysis was performed using Kaluza software.

### Ethical considerations

The published pediatric RISK (GSE101794) and adult SOURCE (GSE199906) treatment naïve CD cohorts were used to test the baseline expression of *LHFPL3-AS2* in CD ileum. Other published cohorts were used for expression validation of *LHFPL3-AS2* in the ileum and colon tissue in healthy controls in bulk biopsies^26^, and in isolated epithelia^26^. Sheba Medical Center Institutional Review Board approved the SOURCE CD cohort protocol and informed consent was obtained.

### Supplementary Information

Supplementary Information 1.

Supplementary Information 2.

Supplementary Information 3.

Supplementary Video 1.

Supplementary Video 2.

Supplementary Video 3.

## Data Availability

mRNAseq generated as part of this study were deposited in GEO: GSE216810. Other datasets used include the RISK (GSE101794) and the SOURCE (GSE199906) cohorts. All authors had access to all data and reviewed and approved the final manuscript.
