# Supplementary material for: Reduced LHFPL3-AS2 lncRNA expression is linked to altered epithelial polarity and proliferation, and to ileal ulceration in Crohn disease
Source: Sci Rep. 2023 Nov 22;13:20513. doi: 10.1038/s41598-023-47997-7 (PMC10665440; doi:10.1038/s41598-023-47997-7)
Supplement: Supplementary file 3 — Supplementary Information 3. [file 41598_2023_47997_MOESM3_ESM.pdf]

**Reduced *LHFPL3-AS2* lncRNA expression is linked to altered epithelial polarity and proliferation, and to ileal ulceration in Crohn disease**

Katya E. Sosnovski[1,2], MS, Tzipi Braun[1], MS, Amnon Amir[1], PhD, Marina BenShoshan[1,2], PhD, Haya Abbas-Egbariya[1,2], MS, Rakefet Ben-Yishay[1], PhD, Liat Anafi[1], MS, Camilla Avivi[1], PhD, Iris Barshack[1,2], MD, Lee A. Denson[3], MD, Yael Haberman[1,2,3], MD, PhD

[1]Sheba Medical Center, Tel-Hashomer, affiliated with the Tel Aviv University, Tel Aviv, Israel.

[2]Faculty of Medicine, Tel Aviv University, Tel Aviv, Israel.

[3]Cincinnati Children's Hospital Medical Center, Department of Pediatrics, University of Cincinnati College of Medicine, Cincinnati, OH, USA.

Corresponding Author:
Yael Haberman, MD, PhD (Yael.Haberman@sheba.health.gov.il and yael.haberman@cchmc.org)
Sheba Medical Center, Tel Hashomer, Israel.
Telephone number: 972-3-5302692

## Supplementary Material

**Supplementary Tables** *– page 3*

**List of supplementary datasets and video files** *– page 4*

**Supplementary Figures** *– page 5*

**Supplementary Methods** *– page 11*

## Supplementary Tables

| **Table S1:** SOURCE treatment naïve cohort | | |
|---|---|---|
| | Ctl<br>(n=12) | CD<br>(n=8) |
| Age (Mean ± SD) | 32 ± 10 | 30 ± 14 |
| Sex M (%) | 6 (50%) | 5 (63%) |
| Disease location<br>L1<br>L2<br>L3 | <br>-<br>-<br>- | <br>4 (50%)<br>0 (0%)<br>4 (50%) |
| Behavior status<br>B1<br>B2<br>B3 | <br>-<br>-<br>- | <br>6 (75%)<br>1 (12%)<br>1 (12%) |

| **Table S2– gRNAs sequences** (5' – 3') | | |
|---|---|---|
| | Sense (Top) | Antisense (Bottom) |
| LHFPL3-AS2 gRNA1 | CACCGAAAGTGGACTACTACCTCAGT | TAAAACTGAGGTAGTAGTCCACTTTC |
| LHFPL3-AS2 gRNA2 | CACCGCTACCTCATGGCATGTTTTGGT | TAAAACCAAAACATGCCATGAGGTAGC |

| **Table S3 - qPCR primers** | | | |
|---|---|---|---|
| Gene | For (5' – 3') | Rev (5' – 3') | Product size |
| LHFPL3-AS2 | CCCGTTTTGCTGCTCACTA | TTGTTTTCCTTAGGCCATCC | 182 |
| CTNNB1 | TGGATACCTCCCAAGTCCTG | CATTAGTGGGATGAGCAGCA | 195 |
| TCF4 | ACTTACAGGGTTGCCACCAG | GCCTCCTTCGGGGATTATT | 129 |
| WT1 | TACAGCACGGTCACCTTCG | CACCGAGTACTGCTGCTCAC | 141 |
| CDH11 | GGTCTGGAACCAGTTCTTCG | GCTCCTTCCCCTGAGAGAAT | 126 |
| SCRIB | ACCATGCTCAAGTGCATCC | AAAAGGCTTGGGCAGCTC | 168 |
| TFRC | GTGCTGTCCAGCAGCCATAG | TCATTCTGAACTGCCACACA | 124 |
| MYO1D | GGGCGCATCTATACGTTCAT | GGCGGTCTCTCATACAGCTC | 122 |
| ZO-1 | CCGAGGGATAGAAGTGCAAG | CCATCTCTTGCTGCCAAACT | 197 |
| GAPDH | TGGACCTCATGGCCCACA | TCAAGGGGTCTACATGGCAA | 169 |
| CDH1 | GAATGACAACAAGCCCGAAT | TCAGGATCTTGGCTGAGGAT | 126 |

**Supplementary datasets**

**Supp. Dataset 1:** LHFPL3-AS2 co-expression and functional enrichment in the RISK cohort

**Supp. Dataset 2:** Differentially expressed genes between LHFPL3-AS2 knockdown and controls Caco-2 cells [fold change differences (FC) >=1.5 and false discovery rate correction (FDR<= 0.05)]

**Supp. Video 1-3**: Z-stack images of 3D cysts with normal bi-polar and abnormal mitotic spindles observed in LHFPL3-AS2 knockdown (video 2-3) and controls (video 1).

## Supplementary Figures

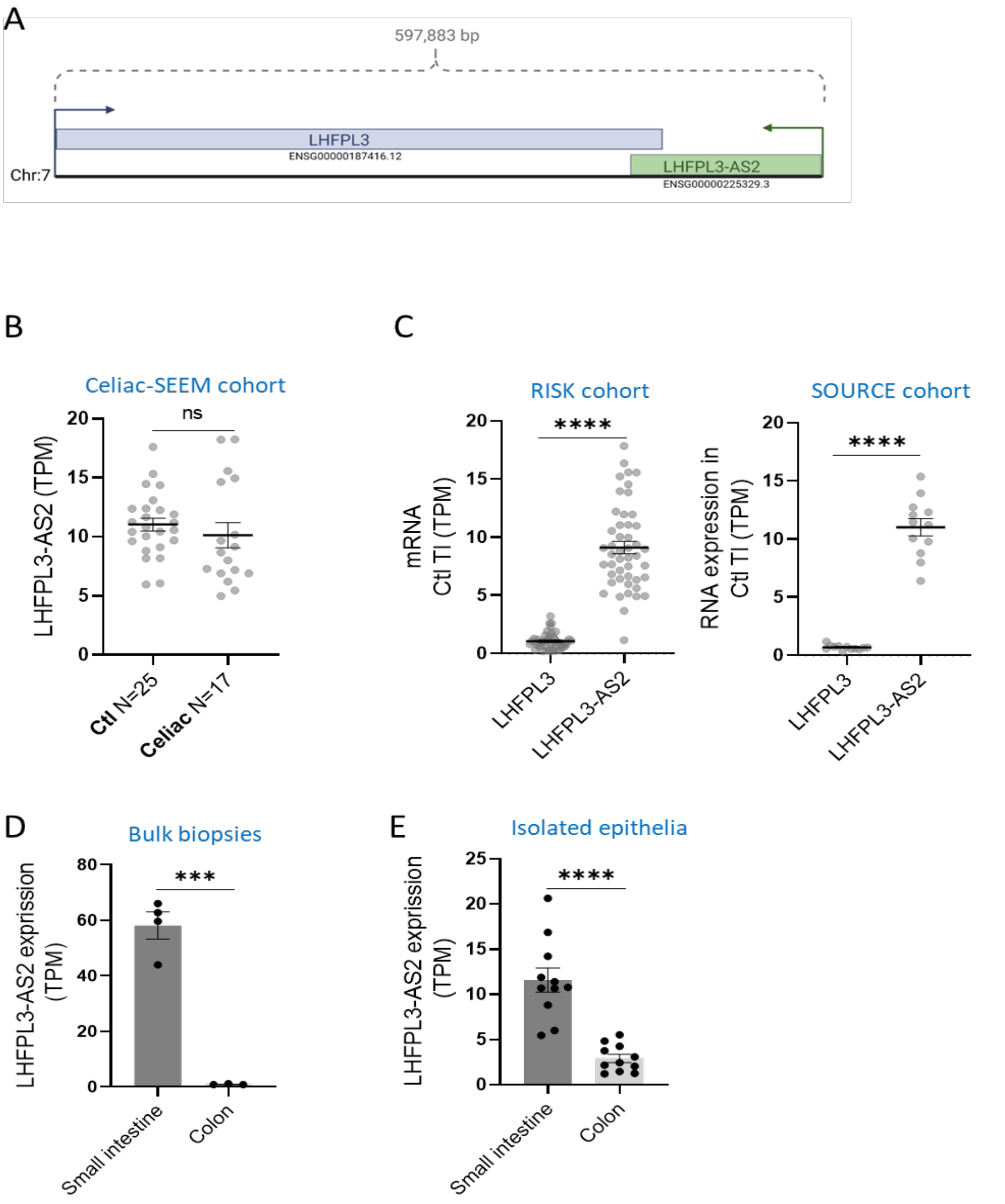


**Fig. S1| LHFPL3 is barely detected in the ileum and LHFPL3-AS2 shows more prominent expression in the small intestine in comparison to the large intestine (colon). A.** Schematic representation of LHFPL3-AS2 genomic locus, showing region human chromosome 7. Green indicates LHFPL3-AS2 and blue indicates LHFPL3. Arrows indicate transcription direction. **B.** LHFPL3-AS2 mRNA expression in duodenum bulk biopsies of controls (n=25) and celiac (n=17) in SEEM cohort, with no significant differences in LHFPL3-AS2 expression in celiac disease **C.** LHFPL3-AS2 and LHFPL3 mRNA expression in terminal ileum bulk biopsies of controls in RISK (n=47) and SOURCE (n=12) indicating minimal or no expression of LHFPL3. **D.** LHFPL3-AS2 levels are significantly increased in bulk biopsies of healthy human small intestines in comparison to colon biopsies. **E.** LHFPL3-AS2 mRNA expression in isolated epithelia from control was more pronounced in the small intestine in comparison to colon tissue. Two-sided t-test is shown. ***P < 0.001, ****P < 0.0001.

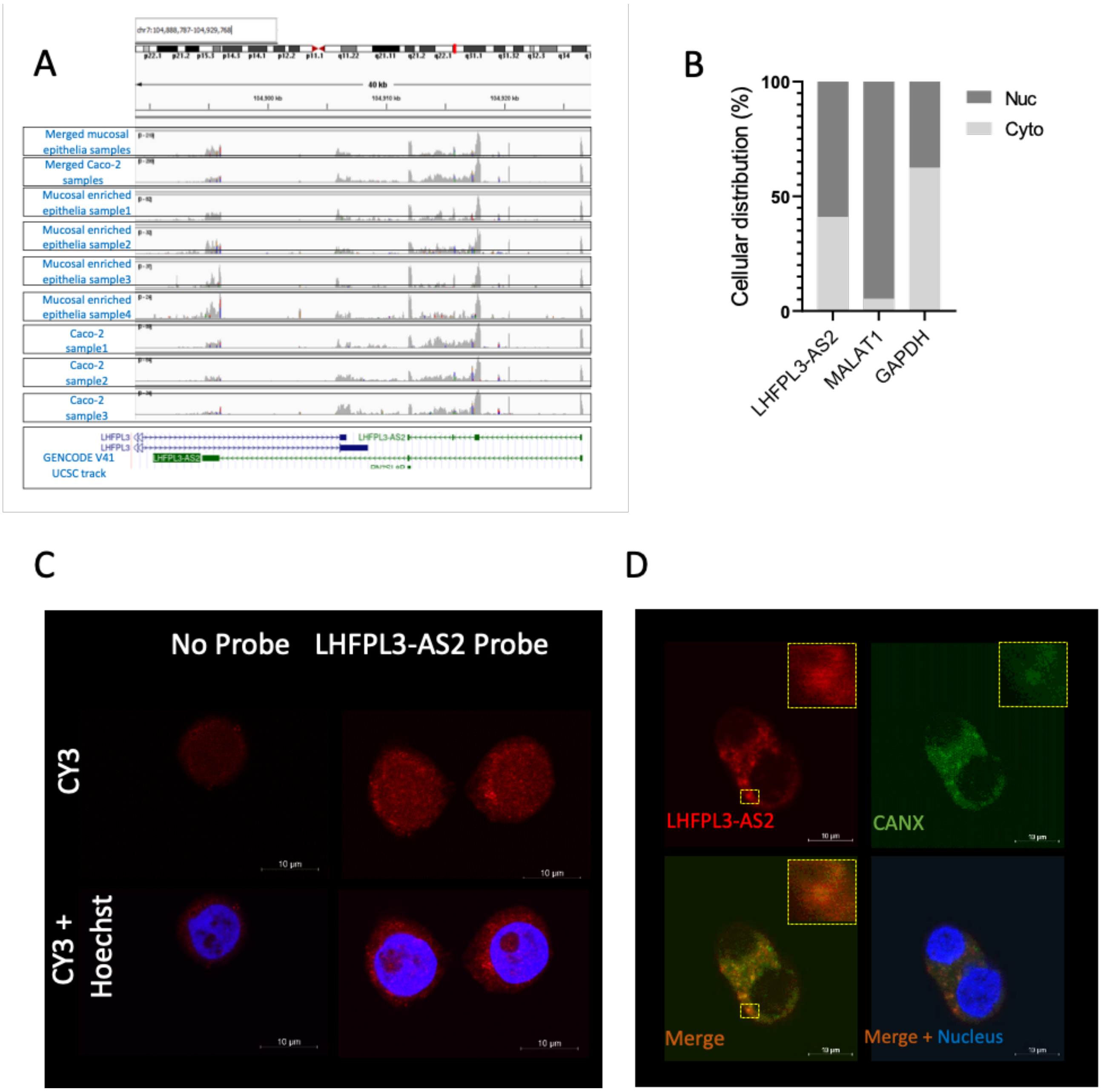


**Fig. S2| LHFPL3-AS2 is detected in the nucleus and cytoplasm showing colocalization with the ER marker CANX in the cytoplasm. A.** LHFPL3-AS2 expression in human mucosal biopsies enriched epithelia and Caco-2 cells, using mRNA-seq. The reads are aligned to genomic region and to the known transcripts from GENCODE V41. The genomic region displayed is chr7:104,888,787-104,929,768. Read visualization using IGV is presented (www.broadinstitute.org/igv/).
**B.** Subcellular detection of LHFPL3-AS2 mRNA in Caco-2 cells indicating distribution between the nucleus and the cytoplasm. MALAT1 is used as a nuclear control and GAPDH as a cytoplasmic control. **C.** Fluorescent in situ hybridization (FISH) shows LHFPL3-AS2 (red) distribution between the nucleus and cytoplasm in Caco-2 cells in comparison to no probe negative control. Nuclei are stained with Hoechst (blue), magnification x63oil, scalebar-10uM. **D.** Immunofluorescence of CANX (green), an ER marker, and RNA-FISH using LHFPL3-AS2 specific probes (red). The colocalization inlet region is presented in the yellow box. Nuclei are stained with Hoechst (blue), magnification x63oil, scalebar-10uM.

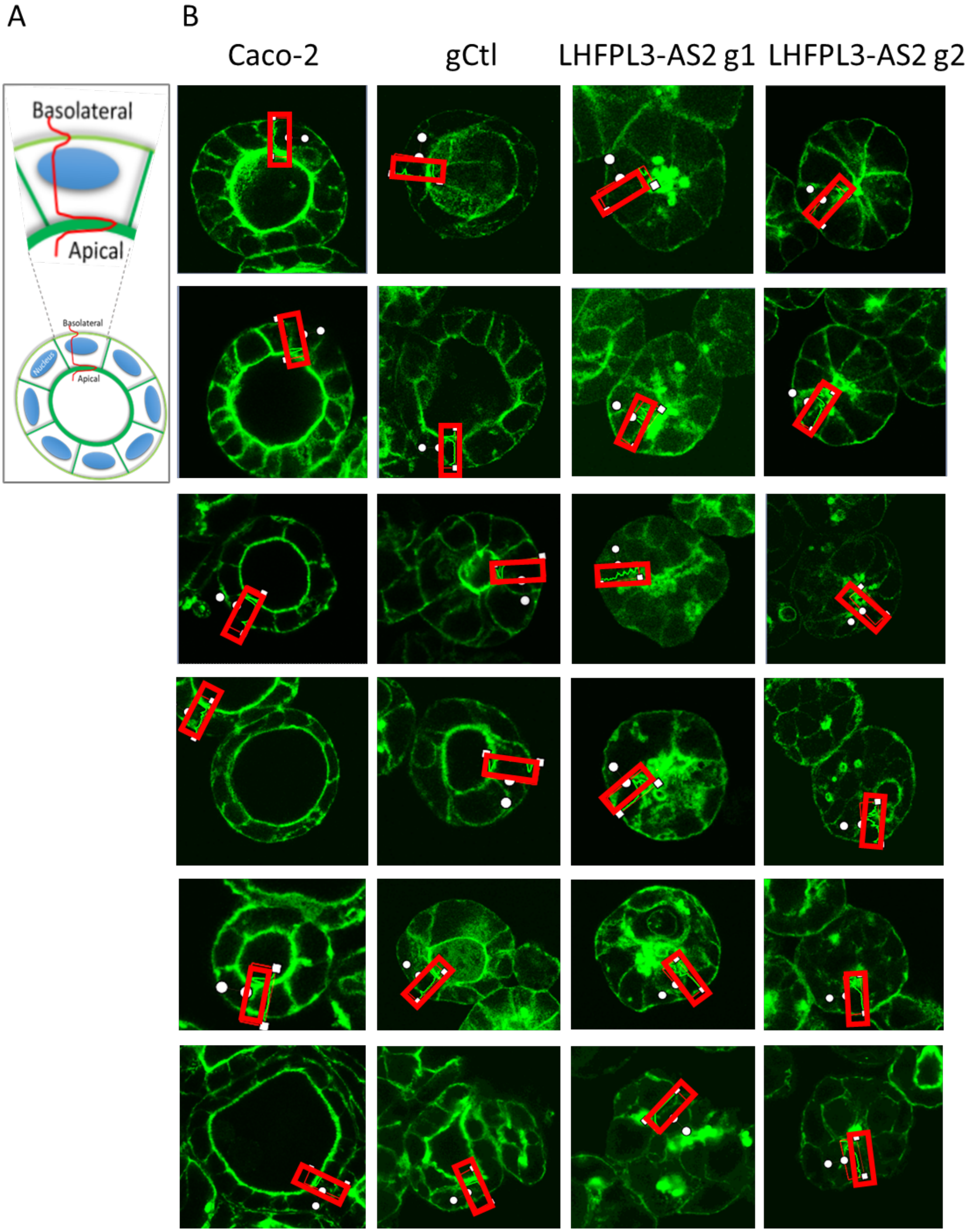


**Fig. S3| LHFPL3-AS2 knockdown disrupts apicobasal actin signal in Caco-2 3D cyst.**
**A.** Schematic representation of the plotted area within a cyst as done in Fig. 2G. The intensity of actin is measured across the indicated red line. **B.** Basolateral and apical actin signal, within the indicated cysts (n=6).

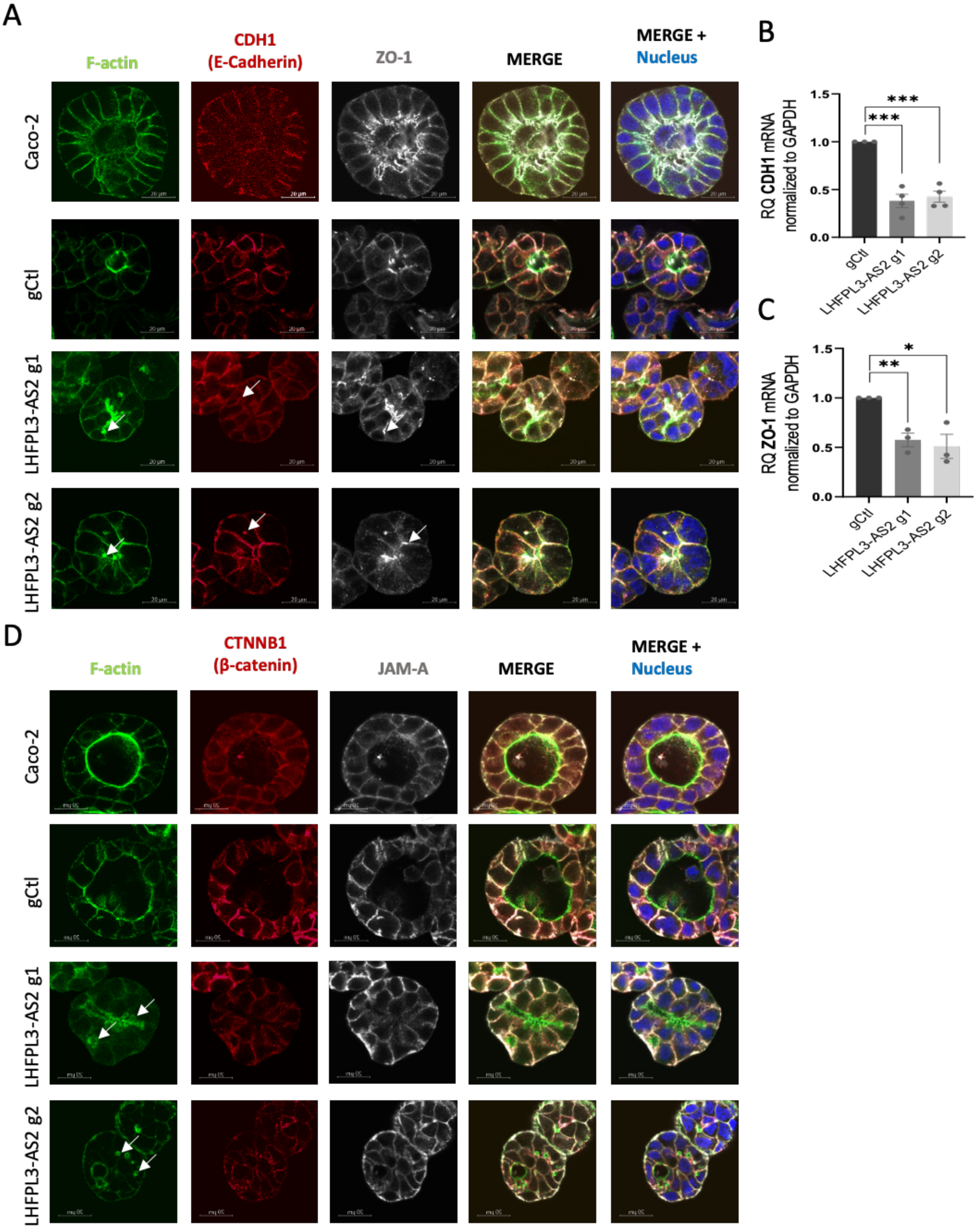


**Fig. S4| LHFPL3-AS2 downregulation resulted in the mislocalization of cell polarity markers.** **A.** Fluorescence staining using phalloidin for F-actin (green), anti-CDH1 (E-cadherin, red) and anti-ZO-1 (grey) of LHFPL3-AS2 knockdown cells (g1 and g2) compared to controls (gCtl) Caco-2grown as cyst in Matrigel using confocal microscopy. Merged image is presented with/without Hoechst (blue) staining. Scalebar – 20um. **B-C.** qPCR of CDH1 and ZO-1 levels as indicated in LHFPL3-AS2 knockdown cells (g1 and g2) and controls (gCtl). qPCR results were normalized to GAPDH. **D.** Fluorescence staining using phalloidin for F-actin (green), anti-CTNNB1 (β-catenin, red), and anti-JAM-A (grey). Merged image is presented with/without Hoechst (blue) staining. Scalebar – 20um. Arrows indicate intracellular inclusions of the indicated marker.

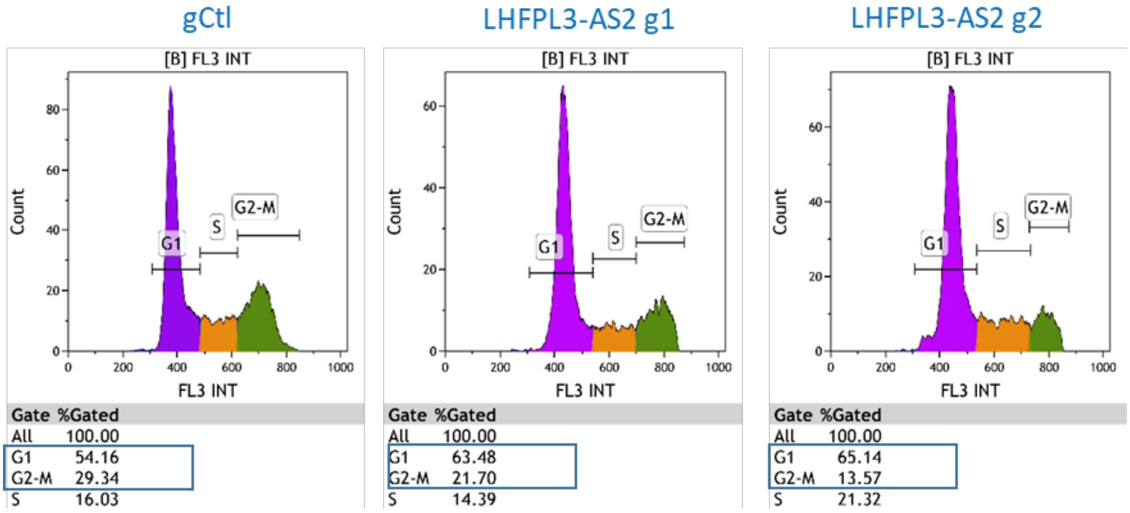


**Fig. S5| Representative Propidium Iodide staining (PI) by FACS analyses of LHFPL3-AS2 knockdown cells (g1 and g2) and controls (gCtl).** Representative images of cell cycle distribution are shown for LHFPL3-AS2 knockdown cells (g1 and g2) and controls (gCtl).

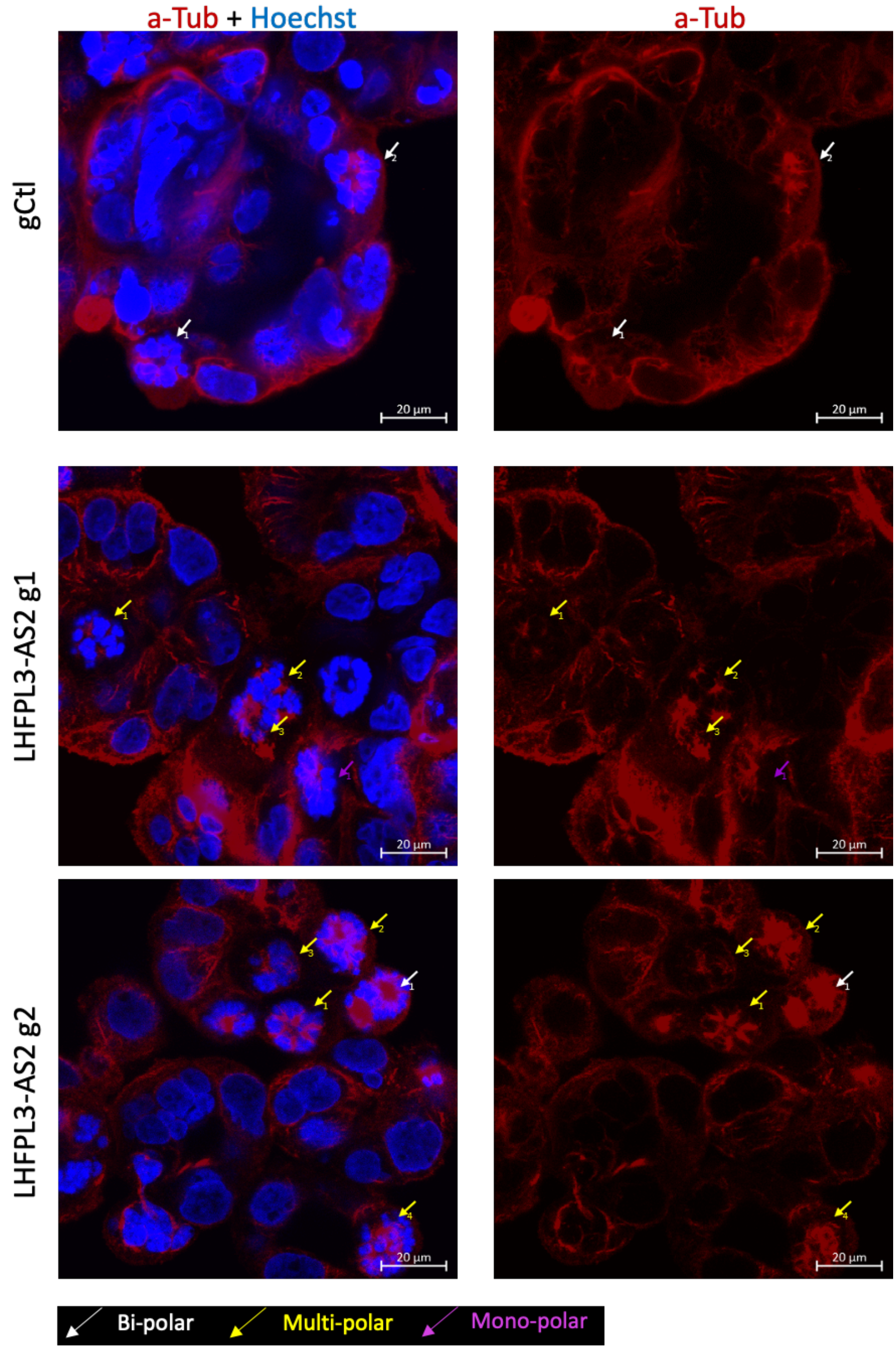


**Fig. S6| Representative images of cells with normal bi-polar and abnormal mitotic spindles observed in LHFPL3-AS2 knockdown and controls.** Cells were cultured in a 3D Matrigel and were treated with 20nM Taxol for 24hr to arrest the cell cycle at mitosis. Representative images for quantification of mitotic spindles (a-Tubulin staining emphasizes the centrosomes and Hoechst stains the nuclei) visualization as bi-polar (white arrows, 2 centrosomes), multipolar (yellow arrows, multiple centrosomes), or mono-polar (purple arrows, one centrosome) as further summarized in Fig. 6C. Scalebar – 20uM, magnification – x60oil. See also Supp. Video 1-3 (The frames generated from 5 Z-stack images of the same region. 5 images/sec, total video length - 5 sec).

A Supplementary material to Fig.3G

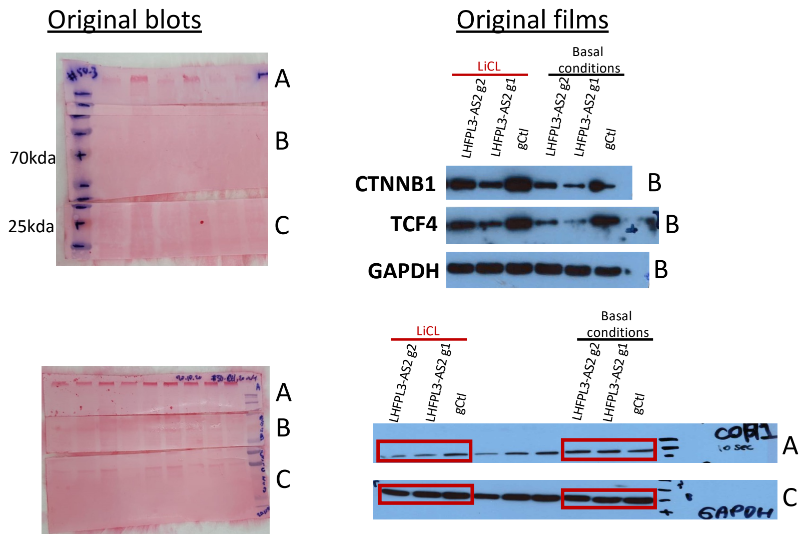


B Supplementary material to Fig. 4B

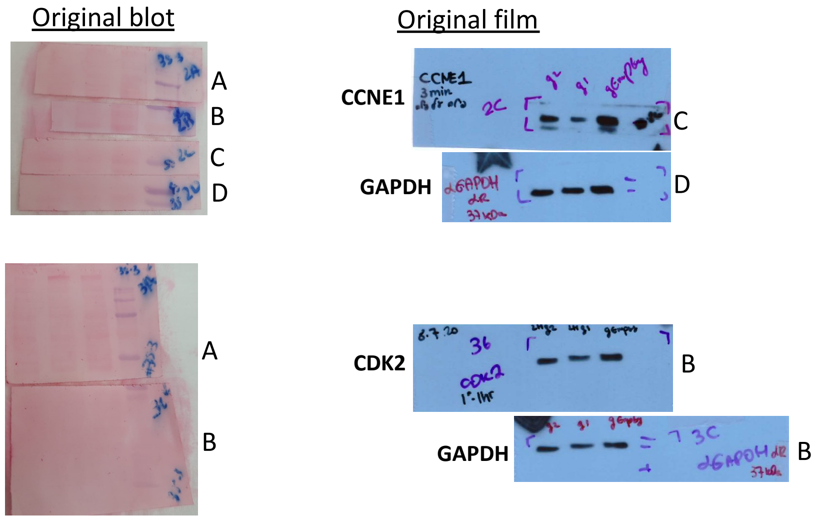


**Fig. S7| Full-length blots of western blot analyses.** full-length blots with Pouceaus S staining, related to the cropped blot's WB images. The blot membranes were cut after the blocking step, to enable incubations with a range of different antibodies at the same time. Each piece of the membrane was marked and can be identified in both images. Blots and films corresponding to blots in Fig. 3G (A) and to Fig. 4B (B).

## Supplementary Methods

**RNAseq datasets.** RISK cohort (GSE101794), a pediatric treatment naïve pediatric CD cohort, and SOURCE (GSE199906) an adult treatment naïve CD cohort were used to test the baseline expression of LHFPL3-AS2 in CD ileum. RISK cohort was previously published in [1-3] and included 213 CD and 47 controls with baseline mucosal ileal biopsies, and the presence of deep ulcers (DU) was recorded during endoscopy. SOURCE included 8 CD and 12 non-IBD controls from the Sheba Medical Center. Age, gender, and endoscopic findings were recorded (Table S1). Sheba Institutional Review Board approved the protocol and safety monitoring plan. Informed consent was obtained for each participant. Transcriptomics analyses started from the raw FATSQ files using a similar pipeline. Reads were quantified by Kallisto [4] version 42.5 using Gencode v24 as the reference genome. Kallisto output files were summarized to gene level using the R package tximport version 1.12.3 [5]. RNA-seq on LHFPL3-AS2 knockdown Caco-2 cells was performed using Lexogen QuantSeq 3' mRNA-Seq libraries sequencing (GSE216810). Principal Coordinates Analysis (PCA) was performed to summarize variations in gene expression between patients. We included 11,489 protein-coding mRNA genes with Reads per Million (RPM) above 3 in 20% of the samples in our downstream analysis. Differentially expressed genes were determined in GeneSpring® software with fold change differences (FC) ≥1.5 and using the Benjamini–Hochberg false discovery rate correction (FDR≤ 0.05).

**Cell culture and organoids.** Caco-2 human colon carcinoma cell line was purchased from the American Type Culture Collection (ATCC, Manassas, VA, USA) and maintained in standard culture conditions in DMEM (GIBCO 41965-039, Scotland) containing 20% (v/v) heat-inactivated fetal bovine serum (GIBCO 12657-029, Scotland). Cells were maintained at 37°C in a humidified atmosphere containing 5% CO2. Caco-2 reached full confluency in all experiments, and the experiments were performed at intermediate differentiation state (day 3-6 after seeding), which showed a reduction of 50% in stemness marker *LGR5* level and induction in the mature enterocytes *APOA1* (200% from baseline) and *SI* (20 times from baseline) levels. Activation of the Wnt/β-catenin pathway was obtained by adding 20mM LiCl to cell media for 24hr, cell lysates were collected for protein extraction. To determine the cellular localization of lncRNAs, cytosolic and nuclear RNA fractions were isolated from Caco-2 cells using the PARIS kit (Ambion, Thermo Fisher Scientific, Stockholm, Sweden) based on the manufacturer's instructions. Briefly, Caco-2 cells were trypsinized, pelleted, and washed in PBS. Then, cells were resuspended in Cell Fractionation Buffer (PARIS kit) on ice and incubated at $4^C$ for 10 min. Lysates were centrifuged at 500 g at $4^C$ for 5 min and the supernatant (cytoplasmic fraction) was transferred to new tubes. Then, the nuclear pellet was washed

in PBS and lysed in an ice-cold Cell Disruption Buffer, with vigorous vortexing. The RNA was isolated by adding a Lysis/Binding solution to each fraction, which was followed by the addition of 100% ethanol and the capture of the RNA by a filter cartridge. The RNA was eluted in a pre-heated Elution solution and stored at -70$^{C}$. The sub-cellular localization was confirmed using RNA-seq of whole cells and in cytoplasm and nucleus fractions. MALAT1 and GAPDH served as markers for the nuclear and cytoplasmic fractions, respectively.

To form 3D cysts, Caco-2 cells were seeded with Matrigel (Corning 354234) and 20% DMEM media (1:1) on 24 well plates. The cells were grown for 6 days to form cysts, the medium was changed every 3 days. To quantify lumen formation, cysts were fixed in 4% buffered formaldehyde, and paraffin-embedded sections were stained with hematoxylin and eosin (H&E). The slides were scanned, and the images were obtained using PMA. start software, then the cysts were counted manually. More than 100 cysts from either control or knockdown cells were examined. For proliferation assessment, paraffin-embedded sections of the cysts were stained with Ki67 antibody.

Crypts isolation and organoids culturing: L-WRN medium (L-WRN media) was generated using the ATCC mouse fibroblasts cells (L-WRN cells - CRL-3276™), that produce Wnt-3A, R-spondin 3, and noggin [6]. To maintain proliferation, TGFBIR inhibitor (SB431542) is added. To initiate differentiation, EP4 inhibitor (L-161,982) is added to DMEM-F12 (Gibco 12634010) medium without FBS. Intestinal biopsies from patients undergoing evaluation via endoscopy according to approval by the Ethics Committees of the Sheba Medical center were used after written informed consents were obtained from patients and/or families. Two biopsies were used for crypt isolation. Biopsies were washed 3 times with PBS and Gentamicin/Amphotericin (X 500- corning), cut into small pieces and incubated in cold for 30 minutes with Gentle Cell dissociation Reagent (STEM CELL 100-0485) that helps to release the crypts. Isolated crypts were seeded in Matrigel (Corning 354234) and L-WRN medium to generate the organoids. Cells were maintained at 37°C in a humidified atmosphere containing 5% CO2 and were passaged weekly. IFNγ (25 ng/ml), and TNFα (20 ng/ml) were applied after differentiation for 48h for an additional 24h in the differentiation conditions.

**LHFPL3-AS2 knockdown.** To generate the CRISPR inactivation (CRISPRi) stable cell line, Caco-2 cells were infected with CRISPR/dCAS9-KRAB inactivator plasmid (addgene #50919) that was packaged into virions generated in HEK293T cells using lentiviral vector system (psPAX2 and pMD2.G plasmids) via Calcium phosphate kit (Promega). Monoclonal clones were selected with Puromycin. dCAS9 expression was confirmed with qPCR and Western Blot analysis that was performed using HA antibody and GAPDH as a loading control. gRNAs directed toward 50-250 bp downstream to TSS (transcription start site) of LHFPL3-AS2 and were designed (Table S2). by:

http://crispr.mit.edu tool. Each gRNA was cloned into a pKLV-hygro plasmid (addgene #50946) and confirmed by sequencing. Then the pKLV-gRNAs clones which contained the U6 promoter and hygromycin gene (hygro) were packaged into virions and generated in HEK293T cells using lentiviral vector system (the vector and psPAX2 and pMD2.G plasmids) with Calcium phosphate kit (Promega). Afterward, a stable clone of Caco-2 expressing CRISPRi plasmid (#50919) was infected by a lentivirus carrying the specific gRNA or with an empty plasmid that is used as a control (gCtl). Infected cells with stable LHFPL3-AS2 expression downregulation and controls underwent selection by hygromycin antibiotics.

**Confocal immunofluorescence.** Cells were grown on coverslips in 12 well plates or Matrigel in 24 well plates for 3D cysts and fixed with 4% paraformaldehyde and permeabilized with 0.1% Triton X-100 for 3 min at room temperature and blocked in 5% BSA for 1 h at room temperature. Then, the cells were incubated with primary antibodies diluted in 5% BSA overnight at 4C. The next day, the coverslips were fluorescently labeled - incubated with a secondary antibody (anti-rabbit or anti-mouse Cy3 or Cy5; Abcam) at a dilution of 1:200 in 5% BSA for 1 h at room temperature. For visualization of F-actin, Phalloidin (dilution 1:1,000 in 5% BSA; Abcam) staining was performed for 20 min, followed by Hoescht staining (5 min at room temperature, 1:1,000 dilution, Sigma). The coverslips were then mounted in Fluoromount-G and examined on a Zeiss confocal microscope (Carl Zeiss AB, Stockholm, Sweden). Images were processed using ZEN 3.1 (blue edition) browser software. For the 3D system, images were produced using a Z-stack confocal-based scan of the cysts. In the case of 3D images, the 3D feature of ZEN 3.1 (blue edition) browser was used. Movies were produced from Z-stack images using the ZEN 3.1 (blue edition) browser. For mitotic spindle visualization, Caco-2 3D cysts were treated with 20nM Taxol for 24hr and stained with a-Tubulin. Mono/bi/multi-polar spindles were quantified manually, across the Z-stack of the cyst. More than 50 mitotic events from either control or knockdown cells were examined. Apical-basal actin intensity was determined, using the profile feature, within the ZEN 3.1 software, which shows intensity to distance curve of the region of interest (luminal apical to the basolateral side of the cyst). Antibodies used for immunofluorescence include – CTNNB1 (ab32572 Abcam), JAM-A (sc-53623 Santa-Cruz), CDH1 (#9782 Cell signaling), ZO-1 (#33-9100 Thermo Fisher), a-Tubulin (ab18251 Abcam). For RNA fluorescent in situ hybridization (FISH), Caco2 cells were seeded on 18 mm glass coverslips in a 12-well plate. The cells were fixed with 1ml of Stellaris fixation buffer (3.7% Formaldehyde, PBS, Nuclease-free water) for 10 min, washed twice with PBS, then stored in 70 % ethanol at 4 °C. The ethanol was removed and 1 ml wash buffer A (20% formamide in Stellaris RNA FISH Wash Buffer A (Biosearch Technologies)) was added. After incubation for 5 min at RT, a 100 µl drop of hybridization buffer (15 % formamide in Stellaris RNA FISH Hybridization Buffer (Biosearch Technologies)) containing

the probe (375 nM) was added onto a parafilm. The coverslip was transferred to the drop with cells facing down and incubated in the dark for up to 16 hr at 37 °C in a humidified chamber. On the next day, the coverslips were transferred to a new 12-well plate containing 1 ml wash buffer A and incubated in the dark at 37 °C for 30 min. For staining the nuclei, the wash buffer was aspirated, and 1 ml Hoechst solution (wash Buffer A consisting of 5 ng/ml Hoechst) was pipetted in each well and incubated for an additional 30 min in the dark at 37 °C. After removing the staining solution, 1 ml wash buffer B (Biosearch Technologies) was added and incubated for 5 min at RT. Confocal imaging was performed with a Carl Zeiss confocal microscope using a 63x oil immersion objective.

**Quantitative PCR (Real-Time RT- PCR) and western blot analyses.** Total RNA was isolated using Tri Reagent-LS (Sigma, T9424, Saint Louis, MO, USA). First-strand cDNA was synthesized using a high-capacity RNA-to-cDNA reverse transcription kit (Applied Biosystems, 4387406). Quantitative real-time polymerase chain reaction (qRT-PCR) was performed using a Fast SYBR Green Master mix (Applied Biosystems, 4385612) and qRT-PCR machine with standard qRT-PCR parameters to analyze the expression of indicated genes compared with the control gene GAPDH. Results were analyzed with the comparative CT method, and log10 (relative quantification values [Rq] values) are shown. All qRT-PCR reactions were performed in triplicates and the statistics were performed on dCt Mean values.  Primers listed in Table S3. For western blot analysis, cells were lysed on ice, in RIPA lysis buffer [R0278-50ML, Sigma] in the presence of a protease inhibitor cocktail (Roche). Equal amounts of protein, as determined by the Bradford assay (B6916, Sigma), were resolved by electrophoresis in SDS 10% polyacrylamide gel and then transferred to a cellulose nitrate membrane (10401383, Tamar). The membrane was incubated with one of the following primary antibodies: anti-TCF4 (#2569 Cell Signaling); anti-CTNNB1 (ab32572 Abcam); anti-CDH1 (#9782 Cell signaling); anti-CDH11 (ab151446 Abcam); anti-JAM-A (sc-53623 Santa-Cruz); anti-ZO-1 (#33-9100 Thermo Fisher); anti-CCNE1 (ab33911 Abcam); anti-CDK2 (sc-163 Santa-Cruz); anti-GAPDH (#2118 Cell signaling). Binding of the primary antibody was detected using an enhanced chemiluminescence kit (XLS142,0250, Cyanagen). Original blots with Ponceau stain and the original films of **Figs. 3G and 4B** can be found in **Fig. S7**.

**Fluorescence-activated cell sorting (FACS) cell cycle analyses.** For propidium iodide (PI) staining, cells were trypsinized, centrifuged, and resuspended in PBS, then fixed by incubating in 70% ice-cold ethanol at 4°C, overnight. After fixation, cells were centrifuged for 4 min at 2000 rpm, and the pellet was resuspended and incubated for 40 min at 4°C in 1 ml of PBS. Then, the cells were centrifuged again and resuspended in PBS containing 5 mg/ml propidium iodide (Sigma) and 50 μg/mL RNase A (Invitrogen). After incubation for 20 min at room temperature, fluorescence intensity

was analyzed using Navios flow cytometer (Beckman Coulter). The analysis was performed using Kaluza software.

**Cell Proliferation and viability (XTT) Assay.** Caco-2 cells were seeded into 96-well plates in triplicates. Then, cell proliferation was assessed using the XTT Cell Proliferation Assay Kit (Biological Industries) following the manufacturer's protocols. Briefly, Activation solution (0.1 ml) was mixed with 5ml XTT reagent, then 50µl of the reaction solution was added to each well at indicated time points (days-1,3,6,7) and then incubated for another 2 hours at 37°C, 5% CO2. Finally, absorbance was measured at the wavelength of 470 nm and 660 nm (Tecan Spectrophotometer). The blank value was subtracted from the sample absorbance.

**Colony formation assay**. Caco-2 cells were seeded (1,000 cells/6 well plate) in triplicates and cultured for 13 days to form colonies. Then, cells were washed twice in PBS and fixed with 100% ethanol. Fixed cell colonies were stained with 10% Giemsa for 15 min, photographed, and counted.

**Summary of statistical tests used.** Pearson was used for correlation and T test was used for between-group analyses. $P < 0.05$, $^{**}P < 0.01$, $^{***}P < 0.001$, $^{****}P < 0.0001$. Fisher exact test was performed for quantification of luminized cysts and mitotic spindle presence between groups. Statistical analyses were performed in GraphPad Prism v9.31.

**Study Approval.** We used already published datasets from RISK. SOURCE was approved by the Sheba Medical center Institutional Review Boards. Informed consent was obtained for all participants.

## References


1 Haberman Y, Tickle TL, Dexheimer PJ, et al. Pediatric Crohn disease patients exhibit specific ileal transcriptome and microbiome signature. *J Clin Invest*. 2014;124(8):3617-3633.

2 Haberman Y, Schirmer M, Dexheimer PJ, et al. Age-of-diagnosis dependent ileal immune intensification and reduced alpha-defensin in older versus younger pediatric Crohn Disease patients despite already established dysbiosis. *Mucosal Immunol*. 2019;12(2):491-502.

3 Haberman Y, BenShoshan M, Di Segni A, et al. Long ncRNA Landscape in the Ileum of Treatment-Naive Early-Onset Crohn Disease. *Inflamm Bowel Dis*. 2018;24(2):346-360.

4 Bray NL, Pimentel H, Melsted P, Pachter L. Near-optimal probabilistic RNA-seq quantification. *Nat Biotechnol*. 2016;34(5):525-527.

5 Soneson C, Love MI, Robinson MD. Differential analyses for RNA-seq: transcript-level estimates improve gene-level inferences. *F1000Res*. 2015;4:1521.

6 VanDussen KL, Sonnek NM, Stappenbeck TS. L-WRN conditioned medium for gastrointestinal epithelial stem cell culture shows replicable batch-to-batch activity levels across multiple research teams. *Stem Cell Res*. 2019;37:101430.
